# Comparative effects of hepatocyte growth factor and tacrolimus on acute liver allograft early tolerance

**DOI:** 10.3389/fimmu.2023.1162439

**Published:** 2023-08-08

**Authors:** Quanyu Chen, Zhiqing Yang, Heng Lin, Jiejuan Lai, Deyu Hu, Min Yan, Zhifang Wu, Wei Liu, Zhehai Li, Yu He, Zhe Sun, Ling Shuai, Zhiping Peng, Yangyang Wang, Sijin Li, Youhong Cui, Hongyu Zhang, Leida Zhang, Lianhua Bai

**Affiliations:** ^1^ Hepatobiliary Institute, Southwest Hospital, Army Medical University, Chongqing, China; ^2^ Key Laboratory of Freshwater Fish Reproduction and Development, Ministry of Education, Laboratory of Molecular Developmental Biology, School of Life Sciences, Southwest University, Chongqing, China; ^3^ Bioengineering College, Chongqing University, Chongqing, China; ^4^ Department of Special Medicine, Shanxi Medical University, Taiyuan, China; ^5^ Department of Orthopedics, Peking University Third Hospital, Beijing, China; ^6^ Department of Radiological Medicine, Chongqing Medical University, Chongqing, China; ^7^ Department of Pathology, Southwest Hospital, Army Medical University, Chongqing, China

**Keywords:** immune tolerance, hepatocyte growth factor (HGF), tacrolimus, liver transplantation, T lymphocytes, immunosuppressants

## Abstract

Allostimulated CD8^+^ T cells (aCD8^+^ T cells), as the main mediators of acute liver rejection (ARJ), are hyposensitive to apoptosis due to the inactivation of death receptor FAS-mediated pathways and fail to allow tolerance induction, eventually leading to acute graft rejection. Although tacrolimus (FK506), the most commonly used immunosuppressant (IS) in the clinic, allows tolerance induction, its use is limited because its target immune cells are unknown and it is associated with increased incidences of malignancy, infection, and nephrotoxicity, which substantially impact long-term liver transplantation (LTx) outcomes. The dark agouti (DA)-to-Lewis rat LTx model is a well-known ARJ model and was hence chosen for the present study. We show that both hepatocyte growth factor (HGF) (cHGF, containing the main form of promoting HGF production) and recombinant HGF (h-rHGF) exert immunoregulatory effects mainly on allogeneic aCD8^+^ T cell suppression through FAS-mediated apoptotic pathways by inhibiting cMet to FAS antagonism and Fas trimerization, leading to acute tolerance induction. We also showed that such inhibition can be abrogated by treatment with neutralizing antibodies against cMet (HGF-only receptor). In contrast, we did not observe these effects in rats treated with FK506. However, we observed that the effect of anti-rejection by FK506 was mainly on allostimulated CD4^+^ T cell (aCD4^+^ T cell) suppression and regulatory T cell (Treg) promotion, in contrast to the mechanism of HGF. In addition, the protective mechanism of HGF in FK506-mediated nephrotoxicity was addressed. Therefore, HGF as a tolerance inducer, whether used in combination with FK506 or as monotherapy, may have good clinical value. Additional roles of these T-cell subpopulations in other biological systems and studies in these fields will also be meaningful.

## Introduction

Liver transplantation (LTx) is a life-saving procedure for end-stage liver disease (ESLD), and donor-specific tolerance induction and suitable use of immunosuppressants (ISs) are essential for achieving success. Tacrolimus (FK506) is currently the most commonly used IS in the clinic (1). It was discovered in 1984 and was first used in the clinic as an IS by Starzl et al. in 1989 (2). Its classic mechanism through IL-2 inhibition is well known (3). Interference through other inflammatory cytokines such as interferon-gamma (IFN-γ) and tumor necrosis factor (TNF)-ɑ has also been reported (4). Although FK506 has been the primary immunosuppressive agent used in multiple organ transplantation since it was initially shown to be more effective in preventing graft loss than other ISs in a clinical trial (1, 5), life-long FK506 treatment increases the incidences of malignancy, infection, and nephrotoxicity, thus limiting its efficacy. As such, identification of more reliable ISs to improve graft outcomes in transplant recipients is urgently needed. In recent years, hepatocyte growth factor (HGF) has attracted attention because of its studies in the field of LTx, in particular, its showing a possibility to go across the CD8^+^ T cell alloimmune barrier (6, 7).

HGF is a multifunctional, pleiotropic protein with mitogenic, motogenic, and morphogenic effects in a wide variety of cells in developing and regenerative livers (8). Based on its recent immunomodulatory effect on xenogeneic rodents and pigs during organ transplantation (9–11), we investigated whether HGF has a similar immunomodulatory role in the DA-to-Lewis allogeneic liver transplant rodents, a well-defined allogeneic acute liver rejection model with a well-done operative procedure of orthotopic liver transplantation (OLT) (namely, allo-OLT here). Taking into account the results of our previous study on xenostimulated CD8^+^ T cell apoptosis induced by HGF-secreting stem cells in a hamster-to-Lewis rodents during hyper-rejection after LTx (1), we herein used the allo-OLT model to compare HGF with FK506, aiming to address whether HGF is more beneficial than FK506 in acute tolerance induction in the allo-OLT rodents and the mechanism interpretation. We chose two types of HGF agents: one is an HGF-like drug (namely, cHGF in the present study) for *in vivo* studies that mainly promotes HGF production in blood after intravenous injection (12), and another is human-sourced recombinant HGF (h-rHGF) obtained from commercial sources for *in vitro* coculture studies with magnetic activated cell sorting (MACS)-purified allogeneic aCD4^+^ and aCD8^+^ T cells with HGFand FK506, respectively, by those we compared the identify differences.

The obtained data indicate that HGF is much more efficacious than FK506 in acute tolerance induction for prevention from liver allograft rejection. Notably, humans (livers) with acute liver rejection (ARJ) show similar expression patterns of CD8 and HGF as allo-OLT rats (livers). To our knowledge, this is the first demonstration of the different proneness of HGF and FK506 in the immunomodulation of T cell subpopulations (CD8, CD4, and Tregs) during acute tolerance induction after LTx.

## Materials and methods

### Animals

Male DA (RT1 n RT2 ^a^) rats which were 8 to 10 weeks old and weighing 150–250 g were used as liver donors, and male Lewis (RT1 ^l^ RT2 ^b^) rats weighing 250–350 g were used as recipients. The animals were purchased from Shanghai Laboratory Animal Research Center, China (http://www.slaccas.com), and housed under specific pathogen-free conditions at the Army Medical University animal facility on a 12-h light/12-h dark cycle for 2 weeks before experimentation. All animal experiments were conducted in accordance with the Animal Research: Reporting of *In Vivo* Experiments guidelines. The institutional ethics committee of Army Medical University approved the experimental protocol (registration number: #SYXK YU 2012–0012).

### Human specimen collection and ethical approval

#### Human blood samples

Blood samples were routinely collected from 75 patients who received FK506 therapy 1 and 3 weeks after LTx at the Department of Hepatobiliary Surgery between January 2012 and December 2017 for analysis of leukocytes, neutrophils, and, in particular, the immune lymphocyte population, the results of which are shown in **Table 1**.

**Table 1:** Data of patients who received tacrolimus (FK506) after LTx.

Age, median (IQR)	1 wk before surgery (n = 75)	Tacrolimus (FK506) therapy (oral, 0.2 mg/kg)		
		Post-LTx		
		n = 75 1 wk	n = 74 3 wk	*p*-value
		48 (22–74)	48 (22–74)	
Sex				
Female	16	16	15	
Male	59	59	59	
Survival state				
Survival	75	74	71	
Death	0	1	3	
Inflammatory cells (10^9^/L)				
Leukocytes	6.0001 ± 4.184 (2.0–7.0)	12.2291 ± 5.3585, <0.05	5.605 ± 2.7670, <0.05	
Neutrophils	4.393 ± 3.665 (1.8–6.3)	10.453 ± 5.018, <0.05	4.346 ± 2.592, <0.05	
Eosinophils	0.22 (0.02–0.52)	0.1693 ± 0.3711, <0.05	0.1673 ± 0.5644	
Immune cells				
Lymphocytes	1.0474 ± 0.524 (1.1–3.2)	1.417 ± 0.691, <0.05	0.727 ± 0.388, <0.05	
Monocytes	2.29 (0.1–0.6)	0.494 ± 0.4111, <0.05	0.3085 ± 0.1811	

Collection of samples: 1 January 2012– 12 December 2017. The bloods were drawn routinely.

#### Human liver samples

We were fortunate to have six archival paraffin-embedded human liver specimens (~1 cm^3^) from patients with ARJ; the samples were histologically confirmed by two independent histopathologists. Six hemangioma liver tissue samples, in which the degree of infiltration was less than 5% (denoted as HA), were used as controls (distal, ~1 cm^3^). Each sample was split into 12 parts and processed for Immunohistochemistry (IHC) and immunofluorescence (IF) analyses at three parts per person. All samples were obtained from the Army Medical University Tissue Bank following a protocol approved by the University’s Institutional Review Board (registration number: Ky2022114).

### Establishment of a DA-to-Lewis allogeneic allo-OLT rat model

We used the three-cuff technique for performing LTx. It was performed by using DA rats as donors and Lewis rats as recipients to establish the allo-OLT rat liver acute rejection model based on Kamada’s two-cuff technique. Briefly, these rats were anesthetized by isoflurane inhalation (in O_2_), and hepatectomized donor livers were flushed with 15 mL of University of Wisconsin (UW) solution (40 units/mL) (13, 14) and stored at 4°C until transplantation. The recipient weight and total surgical time (65.0 ± 13.9 min) were similar among all groups. The suprahepatic vena cava was anastomosed end to end. The portal vein (PV), infrahepatic vena cava (IVC), and bile ducts were anastomosed using the cuff technique. The allo-OLT was performed by skilled microsurgeons, and the mean recipient cold ischemic (anhepatic) time was 35.0 ± 1.3 (SD) min, the warm ischemic time was 0.25 ± 0.05 (SD) min, and the graft ischemic time was approximately 31.0 min. Liver allograft survival was measured as the mean survival time (MST) of recipients in days following LTx. After completion of the operation, the animals’ health status was monitored throughout the subsequent experiments. The technical failure rate of LTx surgery was approximately 10%. After completion of the operation, all transplant recipients were kept in single cages and given *ad libitum* access to a standard pellet diet and water. Each transplant recipient was assigned a numerical code, and the individuals responsible for caring for the animals and analyzing tissues were blinded to the treatment groups. The whole surgical procedure is shown in **Supplementary Figures S1A–C**, and acute allograft rejection was assessed based on Banff’s criteria.

### Banff’s criteria for assessing acute liver allograft rejection

Two experienced board-certified pathologists (YHC and GJD) independently and semiquantitatively assessed the acute rejection status for both patients and rats by determining the rejection activity index (RAI) score (RAIS) and overall or total RAISs (tRAISs) based on Banff’s criteria. To this end, three (humans) or eight (rats) liver sections containing portal veins [PVs (a), boxes], bile ducts [BDs (a), boxes], and vascular endothelia [VEs (c), arrows, mainly marking the central vein part in this study] were selected from each individual, and six subjects were subjected to H&E staining to determine infiltrative cells. Each section contains at least five triads for staining analysis and then graded on a scale of 0 (absence) to 3 (severe) for RAISs. Furthermore, three individual RAISs were added together to derive the overall or total final RAIS (tRAIS) on a scale of 0 (absent) to 9 (severe) (15, 16) as follows: 0 to 2: no rejection; 3: borderline, consistent with allo-OLT in rats or ARJ in humans respectively; 4 to 5: mild; 6 to 7: moderate; and 8 to 9: severe. The H&E-stained tissue sections were graded in a “blinded” fashion by a transplant pathologist (AJD) using the Banff criteria for acute liver rejection (15). The significance of differences between means was ascertained using an unpaired Student’s *t*-test, two-way analysis of variance, or a log-rank test using Prism version 5.00 software; *P* < 0.05 was considered significant.

### Experimental design

Both *in vivo* and *in vitro* experiments were performed. For the *in vivo* animal studies, the rats were divided into two transplantation groups and studied on postoperative day (POD) 7 or 10–12. The transplantation groups were as follows: (1) the Lewis rat-to-Lewis rat syngeneic transplant group (Syn, *n* = 20, *n* = 10/time point)—rats that undergo syngeneic LTx are commonly used as long-term grafts acceptance controls (17). The rats in this group survived for more than 1 year, and they were sacrificed on day 128 in this study, *n* = 20, 10/time point; (2) The DA rat-to-Lewis rat transplantation group (allo-OLT as acute liver rejection group, *n* = 24, 12/time point)—168 rats from the allo-OLT group exhibited rejection within 9–12 days, which is consistent with previous observations (18, 19). The remaining allo-OLT rats were randomly assigned to five group treatments: (1) cHGF (*n* = 48, 24/time point), (2) FK506 (*n* = 48, 24/time point), (3) one neutralizing antibody against cMet treatment before cHGF (*n* = 32, 16/time point), (4) cHGF plus one IGg1 control antibody (*n* = 8) and (5) FK506.plus anti-cMet-Ab (*n* = 8). The rats received 4 μg/kg cHGF (equivalent to the clinical dosage) through intrasplenic injection (20) 2 days before grafting and splenic vein injection during grafting and then intravenous (IV) injections every day for 2 weeks after LTx. cHGF is a drug that has been used clinically in China for heart disease (12) and shown to stimulate endogenous HGF production in the blood after IV injection (12). The FK506 group received 0.2 mg/kg/day FK506 (Prograf, Fujisawa Healthcare; by oral gavage, equivalent to the clinical dosage), the most commonly administered IS agent after LTx (21), at a dosage of 0.2 mg/kg/day, which is 2 days before grafting and once every day for 2 weeks after grafting. The rats received anti-cMet-Ab (2x, 200 ng/animal/20 μL, intrasplenic injection) to block cMet function or control antibody IgG1 (the same concentration as the anti-cMet-AB) through the same way as the cHGF but without IV injection approach. Tissues from liver allografts, recipient spleens, and kidneys were collected from rats in these groups (at least *n* = 6) following euthanasia and used for further analysis of allostimulated T lymphocyte subpopulations (CD3, CD4, and CD8) and regulatory T cells (Tregs and FoxP3/CD25/CD4), the levels of hepatic protein, pro- and anti-cytokine profiles, HGF and cytotoxic kidney injury molecule 1 (KIM-1, also known as TIM-1), etc., via IHC and IF staining.

For the *in vitro* studies, fresh naive splenic CD4^+^ and CD8^+^ T cells and recipients of these cells (aCD4^+^ T cells and aCD8^+^ T cells) were purified by magnetic bead cell sorting (MACS) and analyzed using flow cytometry (FCM). These cells were then stimulated with 0.5%–2% phytohemagglutinin (PHA) and treated with a relatively higher dose of h-rHGF (400 ng/mL) (being published) and FK506 (1–8 ng/mL) (22) for 24 h. The number of expanded clones were counted by investigators blinded to the groups. The counting criteria were based on the clonal size of small (a white arrow), middle (a yellow arrow), and large (a pink arrow) as shown in **Supplementary Figure S3D** (top panels).

### Intrasplenic injection

Before LTx, a surgery was performed for intrasplenic injection based on previously reported studies (23, 24) with slight modifications. Briefly, the animals were placed on the operating table under anesthesia [isoflurane inhalation (in O_2_)], and the fur was shaved along the torso and then sterilized with povidone–iodine until dry. The region just below the ribs was palpated, and an approximately 1.5-cm single incision was made to access the peritoneal cavity and gently explore the spleen. A dry/sterile square of gauze was placed underneath the organ to hold it in place and get ready for injection. The 27-G injection needle was slowly inserted into the spleen (approximately 3 mm, close to the spleen hilum) over a period of 40 s when splenic distension/paling was visible. Notably, needle removal is extremely important, and careful manipulation can completely avoid bleeding when the needle is removed: (1) a piece of sterilized wet gauze was placed over the injection site and used to hold the gauze in place while very slowly withdrawing the needle; (2) after needle removal, the injected area was gently pressed with a sterilized cotton swab for 3 min until the area visibly returned to normal. Finally, the suture and disinfection steps were completed.

### IF and IHC staining

Liver graft, splenic, and kidney tissues were either cryoprotected in 30% sucrose overnight and frozen in optimal cutting temperature medium for IF staining (25) or embedded in paraffin (26) for IHC staining.

#### IF staining

Cryoprotected tissues were cut into serial 5-μm sections on a Leica cryostat (CM1950, Germany), blocked with 5% bovine serum albumin (BSA) for 20 min, and then incubated overnight with primary rat monoclonal antibodies against CD4 and CD8α, a mouse monoclonal antibody against Ki-67, and rabbit polyclonal antibodies against CD25, FoxP3, cMet, and FAS diluted 1:100 in phosphate-buffered saline (PBS) (pH 7.4). Normal mouse and rabbit IgG were used at the same dilution as the primary antibody controls. The slides were then washed three times for 5 min each with PBS after primary antibody incubation. Then, the sections were incubated in the dark with Alexa Fluor 488-conjugated anti-rabbit IgG (green) or Cy3-conjugated (red) anti-mouse IgG secondary antibodies diluted 1:2,000 in 5% normal goat serum (NGS) for 1 h and mounted in fluorescence mounting medium. The mounted slides were photographed under a fluorescence microscope (SMZ25/SMZ18; Nikon, Tokyo, Japan). The number of cells from a quarter area per field—with a sum of four areas per field, six fields from one section, eight sections per animal for six animals per group—was quantified by investigators blinded to the status of each animal and compared with the average value obtained for vehicle-treated animals.

#### H&E staining

Paraffin-embedded tissues were stained with H&E according to routine protocols (27). Briefly, after deparaffinization and rehydration, 5-μm longitudinal sections were stained with hematoxylin solution for 5 min and then dipped 5 times in 1% acid ethanol [1% hydrochloric acid (HCl) in 70% ethanol] and then rinsed in distilled water, stained with eosin solution for 3 min, dehydrated in graded alcohol solutions, cleared in xylene, and then examined using a light microscope. The staining intensity of infiltrating cells around the portal vein (PVs), bile duct (BDs), and vascular region (VEs) was determined based on Banff’s criteria and RAI scores in randomly selected at least five triad fields per section, three (human) or eight sections (animal) at the same tissue level from six subjects in each group.

#### IHC staining for cytokine profiles and functional hepatic proteins

Sections of 5 μm from paraffin-embedded liver graft and kidney tissues were deparaffinized and subjected to antigen retrieval using citrate sodium solution (pH 6.0) in a 100- to 800-W microwave three times for 5 min and cooled at room temperature (RT) for 30 min. Endogenous peroxidase activity was blocked with 1% hydrogen peroxide (H_2_O_2_) for 10 min. The slides were incubated with 3% BSA and 0.5% Triton in Tris-buffered saline (TBS) for 1 h. The tissues were then incubated overnight at 4°C with appropriately diluted primary antibodies (1:400) against HGF; CD8α; the proinflammatory cytokines IL-2, IL-12, TNF-ɑ, and IFN-γ and the anti-inflammatory cytokines IL-4 and IL-10; and the functional hepatic proteins aspartate transaminase (AST), alkaline phosphatase (ALP), albumin (ALB), and KIM-1/TIM-1. The sections were then washed in TBS, incubated with a horseradish peroxidase (HRP)-conjugated secondary antibody for 1 h at RT, developed with 3,3′-diaminobenzidine (DAB) for 5 min, counterstained with hematoxylin, and mounted with neutral balsam. The control sections were incubated without substrate or with medium to which 1 mM levamisole was added. A third control was incubated with DAB alone (28). The positive staining area for each marker in each section was calculated by determining the percentage of the total area exhibiting positive staining (brown color) at a magnification of ×100–400. The density of IHC staining was determined randomly. For the correlation analysis of CD8 and HGF, at least 25 positive areas/fields were randomly selected, six fields/sections, three (human) or eight (animal) sections each; others like cytokines and hepatic proteins, sum of one-sixth area/field (shown as boxes in figures), analysis of all fields, six fields/section, eight sections/animal, total of six animals/group. The stained images were observed under a light microscope (BX41 OLYMPUS, Japan) and analyzed using ImageJ software (National Institutes of Health, Bethesda, MD, USA), and integrated optical density values are presented.

### Evaluation of recipient T cell survival

To assess the proliferation of recipient rat aCD4^+^ and aCD8^+^ T cells in allogeneic liver grafts and recipient’s spleens, we performed double IF staining for Ki-67 within aCD4^+^ or aCD8^+^ T cells. To assess cell death in the tissues, terminal deoxynucleotidyl transferase (TdT) dUTP nick-end labeling (TUNEL) staining (to indicate apoptosis) was performed with the One Step TUNEL Apoptosis Assay Kit according to the manufacturer’s instructions. Briefly, the sections were incubated with 10 g/mL DNase I for 10 min at RT to induce DNA nicking as positive and negative controls were not treated with TUNEL enzyme. Then, the sections were washed three times for 3 min each time with PBS, blocked with 5% BSA for 20 min, and incubated with 50 μL of TUNEL labeling mixture for 60 min at 37°C in a humidified chamber. The slides were washed three times in TBS for 3 min after incubation. Red-stained cells with 4′,6-diamidino-2-phenylindole (DAPI)-labeled nuclei (blue) were considered TUNEL (green)-positive cells. The number of these cells in 200 DAPI+ cells from a quarter region per field (shown as boxes in figures) was counted. Then, with a sum of four fields per section and eight sections/animal, a total of six animals per group were analyzed under an inverted fluorescence microscope (Nikon Eclipse Ti series, Tokyo, Japan) (original magnification, ×400) in a blinded manner.

### MACS bead isolation of recipient splenic aCD4^+^ and aCD8^+^ T cells treated with h-rHGF and FK506 in cultures

CD4^+^ and CD8^+^ T cells were obtained from fresh recipient rat spleens on POD 7 or 10–12 by MACS beads (29). A cell suspension was loaded in a column, which was placed in the magnetic field of a MACS separator to negatively purify the unlabeled cells. According to FCM, the purity of the CD4^+^ cells was greater than 96%, and that of the CD8^+^ cells was greater than 93%. With a total of 1 × 10^4^, the cells were first stimulated with 0.5%–1% PHA and treated with different concentrations of h-rHGF (50–400 ng/mL) and FK506 (0.25–40 ng/mL) in 96-well plates containing 200 μL of serum-free Neurobasal-A medium (Gibco, 10888) for 2–6 h. The cells treated with PHA only were used as controls. In this kind of experiment, we selected the concentrations of h-rHGF as 400 ng/mL and FK506 as 1–8 ng/m, respectively, for further coculture experiments, in which the same number of cells were put in 24-well plates with 300 μL of the serum-free medium for an additional 18–24 h. The response was assessed by determining the number of expanded colonies. The counting criteria were based on the more frequent sphere size appearing during cultures—as small, middle, and large, indicated as white, yellow, and pink arrows, respectively, as shown in **Supplementary Figure S3D**.

### FCM analysis of CD8^+^ T cell activity in blood

cHGF-, FK506-treated, and untreated CD8^+^ T cells (5 × 10^5^–1 × 10^6^) from recipient rat blood were isolated and incubated for 30 min on ice with fluorescein isothiocyanate (FITC)-conjugated rat anti-rat CD8α antibody (1:100). After three washes, the stained cells were incubated with rabbit polyclonal PE-conjugated rat IgG (1:100) for an additional 30 min. After three additional washes, the cells were incubated with 7-AAD (1:100) for 30 min. The percentage of T cells among total cells was then assessed by FCM. Cells incubated with isotype-matched antibodies were used as controls.

### Western blot analysis

Protein lysates were obtained by incubating liver allograft tissues in RIPA buffer with 1 mM sodium orthovanadate and protease inhibitor cocktail for 60 min on ice and cleared by microcentrifugation at 10,000 × *g* for 20 min at 4°C. The supernatant proteins (50 to 100 µg) of each sample were separated on 6%- and 12.5%-gradient SDS-PAGE gel and transferred to a PVDF membrane. Then, the membranes were blocked in TBS/0.1% Tween 20/5% BSA and immunoblotted with an antibody against HGF (1:200). After three washes in TBS/0.1% Tween 20, the membranes were incubated for 1 h at RT with HRP-conjugated anti-rabbit or anti-mouse secondary antibodies (1:5,000), developed with enhanced chemiluminescence reagent (Solarbio, China) system and imaged with X-ray films (LAS MINI 4000, Japan). All experiments were independently repeated three times. The protein expression levels were normalized to the expression level of the housekeeping protein glyceraldehyde-3-phosphate dehydrogenase.

### Quantitative polymerase chain reaction analysis

The mRNA expression of CD8 in liver allografts was measured at POD 7 using a PrimeScript RT reagent kit with gDNA Eraser (TaKaRa, Shiga, Japan, no. RR047A) and TB Green Premix Ex Taq II (TaKaRa, Shiga, Japan, no. RR820A) in accordance with the manufacturer’s instructions (Applied Biosystems). The primer sequences were as follows: β-actin: forward, 5′-CCGCGAGTACAACCTTCTTGC, and reverse, ATACCCACCATCACACCCTG-3′; CD8: forward, 5′-GCTTTTTGCCTTCGAGCTATCG, and reverse, GACTTCGTAGCGTACCTCTGG-3′. Reverse transcription was performed at 37°C for 15 min and 85°C for 5 s. The products were amplified at 95°C for 30 s followed by 40 cycles of 95°C for 5 s and 60°C for 30 s on a Touch Real-Time PCR Detection System (Bio-Rad, CA, USA, CFX96). The data were analyzed using Bio-Rad CFX96 Manager. The expression levels were normalized to the expression level of the housekeeping gene β-actin. We used the comparative Ct (ΔΔCt) method to calculate the relative mRNA expression.

### Analysis of serum cytokine profiles and functional hepatic proteins by ELISA

Blood samples were obtained from the aorta abdominals under short-term inhalation anesthesia on POD 7 and 10–12 after LTx using standard enzyme-linked immunosorbent assay (ELISA) (30) following the manufacturer’s instructions to measure the levels of the hepatic proteins AST, total bilirubin, ALP, and ALB; proinflammatory cytokines IL-2, TNF-α, and IFN-γ; and anti-inflammatory cytokine IL-10.

### Coimmunoprecipitation and immunoblotting

The binding between Fas and c-Met on naive splenocyte-purified CD8^+^ T cells was evaluated by co-IP and IB (31). A total of 1 × 10^6^ of these cell lysates were immunoprecipitated with an antibody against either Fas (IP) or cMet (IP) and then subjected to IB with either an anti-cMet-Ab to detect cMet (145 kDa) or an anti-Fas antibody to detect Fas (35 kDa). Briefly, equal amounts of cell lysates (500 µg to 1 mg) were incubated with protein A/G magnetic beads in IP lysis buffer at RT for 2 h and washed three times with 0.1% Tween 20 in TBS for 3 min. Then, protein A/G magnetic beads were incubated with either a mouse IgG1 isotype control antibody (2 µg/mL), a rabbit IgG isotype control antibody (2 µg/mL), or a mouse anti-cMet monoclonal or rabbit polyclonal anti-Fas antibody (2 µg/mL) for 1 h at RT. The protein A/G magnetic beads were centrifuged at 10,000 × g and washed three times with lysis buffer. The pellet was resuspended in Western blot loading buffer and heated at 95°C for 5 min. After centrifugation, the supernatant was resolved on 6% or 12.5% sodium dodecyl sulfate–polyacrylamide gel electrophoresis (SDS-PAGE) gel for western blot analysis.

### Localized surface plasmon resonance spectroscopy of metallic nanoparticles for the analysis of chemical and biological sensing

Localized surface plasmon resonance (LSPR) is a powerful technique for the analysis of chemical and biological sensing (32) and protein–protein interactions (33). In this study, we used this method to deduce whether FAS antagonized by cMet on active aCD8^+^ T cells in allo-OLT recipient rats can be abrogated by HGF (in the form of cHGF) and whether the cHGF treatment induces FAS-mediated apoptosis of recipient aCD8^+^ T cells recruiting Fas-associated protein with death domain (FADD) and caspase-8. Briefly, 50 μg/mL Fas antibody was immobilized on a COOH sensor chip that was activated by injection of 1-ethyl-3-(3-dimethyl propyl)-carbodiimide and N-hydroxysuccinimide at a flow rate of 20 μl/min in running buffer. Free activated carboxyl groups were inactivated by the addition of 100 μl blocking buffer. The immobilized protein was washed with running buffer until a stable baseline was achieved. Total protein (50 μg/mL) isolated from cell lysates was injected into the flow at a rate of 20 μl/min, Fas protein was captured by the sensor, and 50 μg/mL IgG1 was used as a negative control. Antibodies against cMet, FADD, and caspase-8 were added to the sensor chip to assess the interactions of these target proteins with Fas for 5 min, and dissociation was evaluated for an additional 7 min. The kinetic parameters of the binding reactions were calculated and analyzed using Trace Drawer software (Ridgeview Instruments, Uppsala, Sweden).

### Whole-slide imaging and image acquisition

Whole-slide images (WSIs) of liver tissues were obtained with a multicolor automatic digital slide scanner (34), and the images were analyzed with algorithms (35) using ZEN 2.3 (blue edition) software (Carl Zeiss Microscopy GmbH 2011). This software allows the user to navigate images and perform geometric and quantitative measurements. After scanning the liver sections with an Axio Scanner, five regions of interest (ROIs) per slide (images were taken at full resolution with a ROI of 1,364 × 1,364 pixels and pixel at 4.55 µm × 4.55 µm) were selected randomly from across the entire digital slide, with the centrilobular vein being in the center of each ROI, for a total of 250 ROIs. The area of each ROI was quantified. A series of analyses were performed for these 250 ROIs by different investigators. The images were subsequently analyzed using in-house macros in batch mode in ImageJ® software (version 1.53e, National Institutes of Health, USA).

### Iodine I-125 FasL labeling

Labeling with 125I-FasL was performed according to the manufacturer’s instructions (Iodobeads, Knowledge Management, and Development Bioscience, Tianjin, China). FasL protein (5–10 μg) was placed in a siliconized Eppendorf tube containing 100 µl of 100 mM sodium phosphate reaction buffer (pH 6.8) and 0.5 mCi of sodium ^125^I-FasL (Knowledge Management and Development Bioscience, Tianjin, China) and incubated for 8–15 min. The specific activity of ^125^I-FasL was approximately 5 × 10^4^ corn/ng. Briefly, the ^125^I-FasL reaction mixture was then added to duplicate wells at the desired concentration in a total of 1 ml of binding buffer with or without h-rHGF and loaded onto a 5-ml G-25 desalting column in cold binding buffer containing Neurobasal-A medium (catalog no. 10888022, Thermo Fisher, USA), 25 mM N-2-hydroxyethylpiperazine-N′-2-ethanesulfonic acid (catalog no. 15630080, Thermo Fisher, USA), and 1% BSA (catalog no. A8010, Solarbio, China). Splenic aCD8^+^ T cells were isolated from recipient rats using MACS, treated with either cHGF or FK506 and stimulated with 0.5–2% PHA as described above, washed two times with cold buffer, and solubilized with 1 mL of 1% Triton X-100 to extract receptor-associated ^125^I-FasL. Radioactivity was measured with a gamma scintillation counter (PerkinElmer, TRI-CARB 4810TR, MA, USA). Nonspecific binding was determined in the presence of 5–0 μg of pure unlabeled FasL (unless otherwise indicated, all the binding data were corrected for nonspecific binding) (36).

### Evaluation of calcineurin inhibitor toxicity in the kidneys

Sections of 5 μm from recipient kidney tissues were fixed and stained with H&E to visualize renal lesion by CNIT scores (37) under light microscopy, whose diagnostic features of tubular isometric vacuoles (tv, i), tubular atrophy with a striped interstitial fibrosis (ii) and subendothelial, medial, and peripheral arteriolar hyalinosis (ah, iii) were identified. The CNIT score was determined by semiquantitatively grading the degree of these three features (scale 0 to 3), with a total score of 9 in tissues where epithelial necrosis, loss of the brush border, cast formation, and tubular dilation were present. A five-point scale was used to assess tubular necrosis in the kidneys (0: normal; 1: damage to 1%–25% of tubule cells; 2: damage to 26%–50% of tubule cells; 3: damage to 51%–75% of tubule cells; and 4: damage >50%) (37) (**Table 2**). Moreover, the kidney sections were subjected to IHC staining for KIM-1; a kidney injury marker (38) is present in tubule cells (39). A rat monoclonal antibody was used for staining, and KIM-1 expression in each section was determined by selecting the expression of positively stained tubule cells in six random-field pictures taken from one section, eight sections/animal, for a total of six animals in each group, at a magnification of ×200. All information about the antibodies and kits used in this study is provided in **Table 3**.

**Table 2:** Calculation of CNIT scores based on three histologic features of CNIT^1^.

Diagnostic feature	Extent	Score (0 to 3)	Syn	OLT	FK506	cHGF
(i) Tubular isometric vacuolization	None	0	√	√		√
	1 to 25%	1				
	26 to 50%	2			√	
	>50%	3				
(ii) Striped interstitial fibrosis	0 to 5%	0	√	√		√
	6 to 25%	1				
	26 to 50%	2			√	
	>50%	3				
(iii) Peripheral or medial ah	None	0	√	√		√
	10% or fewer arterioles	1			**√**	
	11 to 30% of arterioles	2				
	>30% arterioles	3				
Total score		9				

^1^CNIT, calcineurin inhibitor toxicity.

**Table 3:** Antibodies/reagents/kits.

Item type	Item name	Cat. number	Application(s)	Company	City/state	Country
Primary antibodies	Rabbit CD3e	bs-4815R	Immunofluorescence	Bioss	Beijing	China
	Rabbit CD4	bs-0647R	Immunofluore scence			
	Rabbit CD8	bs-22852R	Immunofluorescence			
	Mouse Ki-67	550609	Immunofluorescence	BD biosciences	California	USA
	Rat CD25	202103	Immunofluorescence	Biolegend	California	USA
	Rabbit Foxp3	bs-10211R	Immunofluorescence	Bioss	Beijing	China
	IL-2	bs-1191R	Immunohistochemistry			
	IL-12	bs-0767R	Immunohistochemistry			
	TNF-α	bs-10802R	Immunohistochemistry			
	IFN-γ	bs-0480R	Immunohistochemistry			
	IL-2	CSB-E04628r	ELISA	Cusabio	Houston	USA
	TNF-α	CSB-E11987r	ELISA			
	IFN-γ	CSB-E04579r	ELISA			
	Rabbit IL-10	bs-0698R	Immunohistochemistry	Bioss	Beijing	China
	Rabbit HGF	bs-1025R	Western blot analysis, immunohistochemistry			
Secondary antibodies	HRP-Labeled Goat Anti-Mouse IgG (H+L)	ZB-2304	Western blot analysis	ZSGB-BIO	Beijing	China
	HRP-Labeled Goat Anti-Rabbit IgG (H+L)	ZB-2305	Western blot analysis	ZSGB-BIO	Beijing	China
	Alexa Fluor® 488 AffiniPure Goat Anti-Mouse IgG (H+L)	115-545-003	Immunofluorescence	Jackson ImmunoResearch	PA	USA
	Cy™3 AffiniPure Goat Anti-Mouse IgG (H+L)	115-165-003	Immunofluorescence			
	Alexa Fluor® 488 AffiniPure Goat Anti-Rabbit IgG (H+L)	111-545-003	Immunofluorescence			
	Cy™3 AffiniPure Goat Anti-Rabbit IgG (H+L)	111-165-003	Immunofluorescence			
	Alexa Fluor® 488 AffiniPure Donkey Anti-Goat IgG (H+L)	705-545-147	Immunofluore scence			
	Cy™3 AffiniPure Donkey Anti-Goat IgG (H+L)	705-165-003	Immunofluore scence			
	Alexa Fluor® 488 AffiniPure Goat Anti-Rat IgG	112-545-006	Immunofluore scence			
Kits	One-step TUNEL Apoptosis Detection Kit (Green Fluorescence)	C1086	Immunofluore scence	Beyotime	Shanghai	China
	DAB Kit	ZLI-9017	Immunohistochemistry	ZSGB-BIO	Beijing	China
	ELISA Kit	bs-3977R(AST)	ELISA	Bioss	Beijing	China
		JL-T1398(TBil)	ELISA	Laibio	Shanghai	China
		JL13545(ALB)	ELISA	Laibio	Shanghai	China
		bs-1191R(IL-2)	ELISA	Bioss	Beijing	China
		CSB-E11987r(TNF-α)	ELISA	Cusabio	Houston	USA
		bs-0480R(IFN-γ)	ELISA	Bioss	Beijing	China
		CSB-E04595r(IL-10)	ELISA	Cusabio	Houston	USA
	Co-IP Kit	3127(cMet)	Co-IP	Cell Signaling Technology	Massachusetts	USA
		13098-1-AP (Fas)	Co-IP	Proteintech	Wuhan	China
	MACS Isolation Kit	CD4	558131	BD Biosciences	California	USA
		CD8	558471	BD Biosciences	California	USA
	Enhanced BCA Protein Assay Kit	P0010	Western blot analysis	Beyotime	Shanghai	China
	RNeasy Mini kit	74194	RT-qPCR	QIAGEN	Hilden	Germany
	PrimeScriptTM RT reagent kit	RR047A	RT-qPCR	TaKaRa Biotech Inc.	Shiga	Japan

### Statistical analysis

All summary data for the different groups are expressed as the mean ± SD of at least three independent experiments, and representative images are also presented. Unpaired *t*-test was used for statistical analyses, and differences were considered significant when the *p*-value was <0.05. For multiple comparisons, individual comparisons of group means were made by one-way analysis of variance (ANOVA) followed by Dunnett’s test using the SPSS 13.0 software package (SPSS Inc., Chicago, IL, USA). When quantifying the cellular composition of cultures, at least three trials were performed in duplicate for each group of cells in each experiment. Linear regression analysis was used to determine the correlation between two parameters. Group means and standard deviations were calculated for each parameter. All statistical analyses were performed with Prism 5.00 (GraphPad Software, La Jolla, CA, USA).

## Results

### Parameters of inflammatory cellular infiltration after LTx in patients who received FK506 therapy

**Figure 1:**
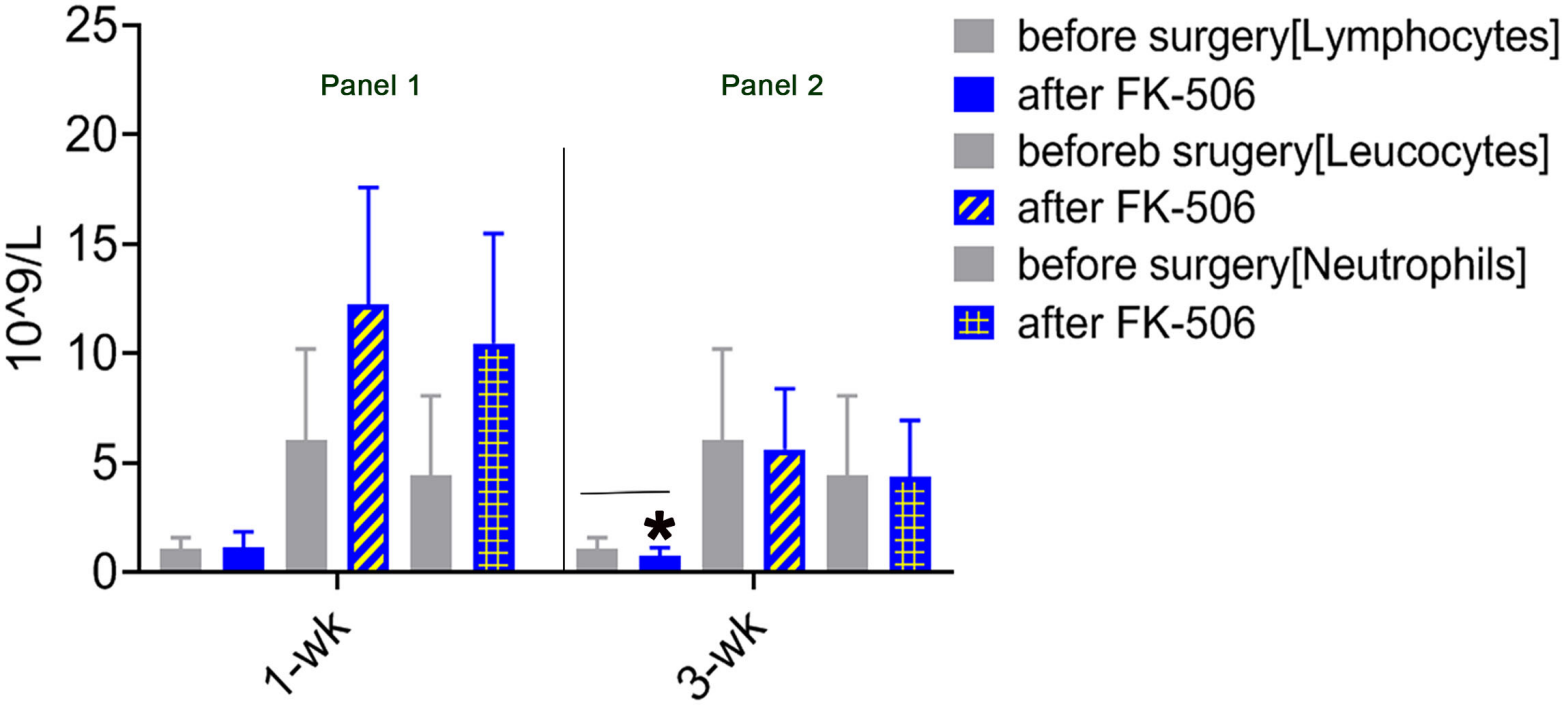
Mononuclear cell infiltration at 1 and 3 weeks after LTx compared with 1 week before surgery in patients who received FK506 therapy (panels 1 and 2). Clinical routine blood analysis in numbers of leukocytes, neutrophils, and lymphocytes at 1 week (panel 1) and 3 weeks after surgery compared with 1 week before surgery (panel 2) in 75 patients who underwent LTx. *p < 0.05 vs. 1 week before surgery.

The immunomodulation effect of FK506 during tolerance induction after LTx has been studied in animals (40). To determine whether the effects observed in animals reflect those in humans, we selected 75 patients who received FK506 therapy post-LTx and analyzed the proportions of not only key inflammatory cells (leukocytes neutrophils) 1 and 3 weeks after FK506 administration but also, for the first time, the proportions of lymphocyte population (**Table 1**). As expected, the proportions of leukocytes and neutrophils were dramatically increased at 1 week after FK506 treatment compared with those at baseline (1 week before surgery) (**Figure 1**, panel 1), and these cells returned to normal after 3 weeks (**Figure 1**, panel 2). However, when analyzing lymphocytes, we found that the proportion of these cells was significantly decreased 3 weeks post-FK506 compared with the baseline (**Figure 1**, panel 2, *p < 0.05). These findings suggest that the early systematic inflammation (1 week post-LTx) may not be due to FK506 in this stage but may be due to lymphocytes, although which lymphocyte subpopulation remains unclear. These preliminary clinical outcomes provide new information about the correlation between FK506 and lymphocyte population that would be clinically meaningful.

### Differences in the effects of cHGF and FK506 in regulating the proportions of infiltrating cells and total CD3+ T cells on POD 7 and 10–12 in the allo-OLT rat model

**Figure 2:**
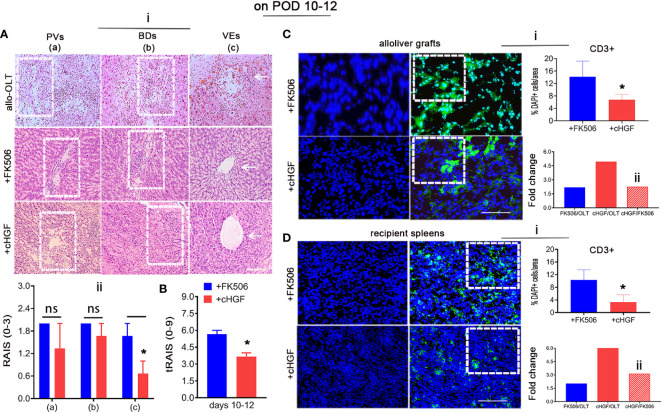
Different effects of cHGF and FK506 in regulating the proportions of infiltrating cells and total CD3^+^ immune T cells on POD 10-12 in allo-OLT model rats. **(A**—i, i-B**)** H&E staining (i) and analysis to identify infiltrating cells around the PVs (a, boxes), BDs (b, boxes), and VEs (c, arrows) in subgroups were performed; original magnification = ×200; scale bar = 100 µm; *n* = 6. Assessment of the rejection grade based on Banff’s criteria via the calculation of the RAI scores [RAISs (0–3) and tRAISs (0–9)]. The cHGF-treated recipient rats(red bars) compared with the FK506-treated rats (blue bars) showed no significant difference in RAISs around PVs and BDs but not VEs (ii), while the calculation of the tRAIS showed dramatic differences in all three parameters **(B)**. **(C, D**—i–ii**)** Detection of CD3+ T cells (green) in liver allograft **(C)**, boxes) and the recipient’s spleen **(D)**, boxes) sections from the cHGF (red) and FK506 (blue) groups by immunofluorescence staining, quantitative analysis (i) and fold changes [the FK506 (blue) and cHGF (red) groups relative to the allo-OLT group (bottom charts); the cHGF group relative to the FK506 group (ii, red striped bars)]; the boxes represent a quarter area per stained field. Original magnification = ×400; scale bar = 200 µm; *n* = 6/group. The data are presented as the mean ± SD of at least three independent experiments. *p < 0.05 vs. the FK506 group. NS, Non-significant.

HGF has also been reported to have suppressive effects on CD8^+^ T lymphocytes (41, 42). To compare the effects of the two reagents in allogeneic LTx recipients, we used the established allo-OLT liver rejection rat model based on Kamada’s two-cuff technique (**Supplementary Figures S1A–C**) assessed on the basis of Banff’s criteria (Mat/Met) and treated the rats with the HGF-promoting drug (cHGF) (Mat/Met) or FK506. H&E staining for infiltrating mononuclear cells around the PVs (a, boxes), BDs (b, boxes), and VEs (c, arrows) demonstrated that, on POD 7, the average RAIS of allo-OLT model rats was approximately 1.5–2.5 (**Supplementary Figure S1E**—i, ii, *p < 0.05), and the average tRAIS was approximately 4.1–4.3 (**Supplementary Figure S1F**), compared with that of the Syn group [**Supplementary Figures S1D** (top panels), E—ii, F; *p < 0.05). On POD 10–12, the average RAIS of the allo-OLT model rats was approximately 2.8–3.18 (**Supplementary Figure S1E**—iii, iv, **p < 0.001), and the average tRAIS was approximately 4 –8.8 (**Supplementary Figure S1F**, **p < 0.001), indicating that allogeneic liver rejection occurred in the model rats. Compared with the allo-OLT group (**Figure 2A**i, top panels) on POD 10–12, both the FK506 (**Figure 2A**i, middle panels) and cHGF (**Figure 2A**i, bottom panels) groups showed a significantly improved inflammation based on the RAI scores (not shown). In a comparison with FK506 (blue bars), cHGF (red bars) showed almost equal effects on the average RAISs around the PVs and BDs (**Figure 2A**i, left two panels, boxes, ii, ns *p* > 0.05). When observing the inflammation around VEs (**Figure 2A**i, right bottom two panels, arrows), we found that cHGF significantly decreased the number of infiltrating cells compared with FK506 (**Figure 2A**ii, *p < 0.05). However, upon analyzing the scores of tRAIS, cHGF showed significant (*p < 0.05) effects on the tRAIS in all areas compared with FK506 (**Figure 2B**), suggesting that cHGF induced a greater pathological improvement than FK506, especially around the injured blood vessels. For lymphocytes, the proportion of total T cells was also significantly decreased in both liver allografts (**Figure 2C**) and recipient spleens (**Figure 2D**) from the rats in the cHGF monotherapy group compared with those from the rats in the FK506 group, as indicated by IF staining with an antibody against CD3 (green). Approximately 6.25% ± 1.77 of CD3^+^ T cells in liver allografts (**Figure 2C**i; the boxes indicate a quarter of field) and 3.27% ± 2.35 of these cells in recipient spleens (**Figure 2D**i, boxes) were found in the cHGF group, while 14.13% ± 5.04% of cells in grafts and 10.31% ± 2.35 of CD3^+^ T cells in the recipient’s spleens were found in the FK506 group; compared with FK506 (blue bars), cHGF (red bars) decreased significantly (**Figures 2C**i, **D**i, *p < 0.05) and induced an almost 2.26 times greater decrease of cHGF over FK506 in the proportion of CD3^+^ T cells in liver allografts (**Figure 2C**ii, striped bar) and a 3.14-fold greater decrease in recipient spleens (**Figure 2D**ii, striped bar), although both FK506 and cHGF induced a greater decrease in the proportion of these cells than allo-OLT alone (not shown), suggesting that the HGF-promoting drug cHGF has a more robust effect on T lymphocytes than FK506. These observations indicate that, at early time points (prior to 3 weeks, POD 10–12), cHGF exerts a superior effect over FK506 on anti-inflammatory and T lymphocyte suppressive effect.

### cHGF mainly affects the proportion of aCD8^+^ T cells while FK506 mainly affects the proportion of aCD4^+^ T cells

**Figure 3:**
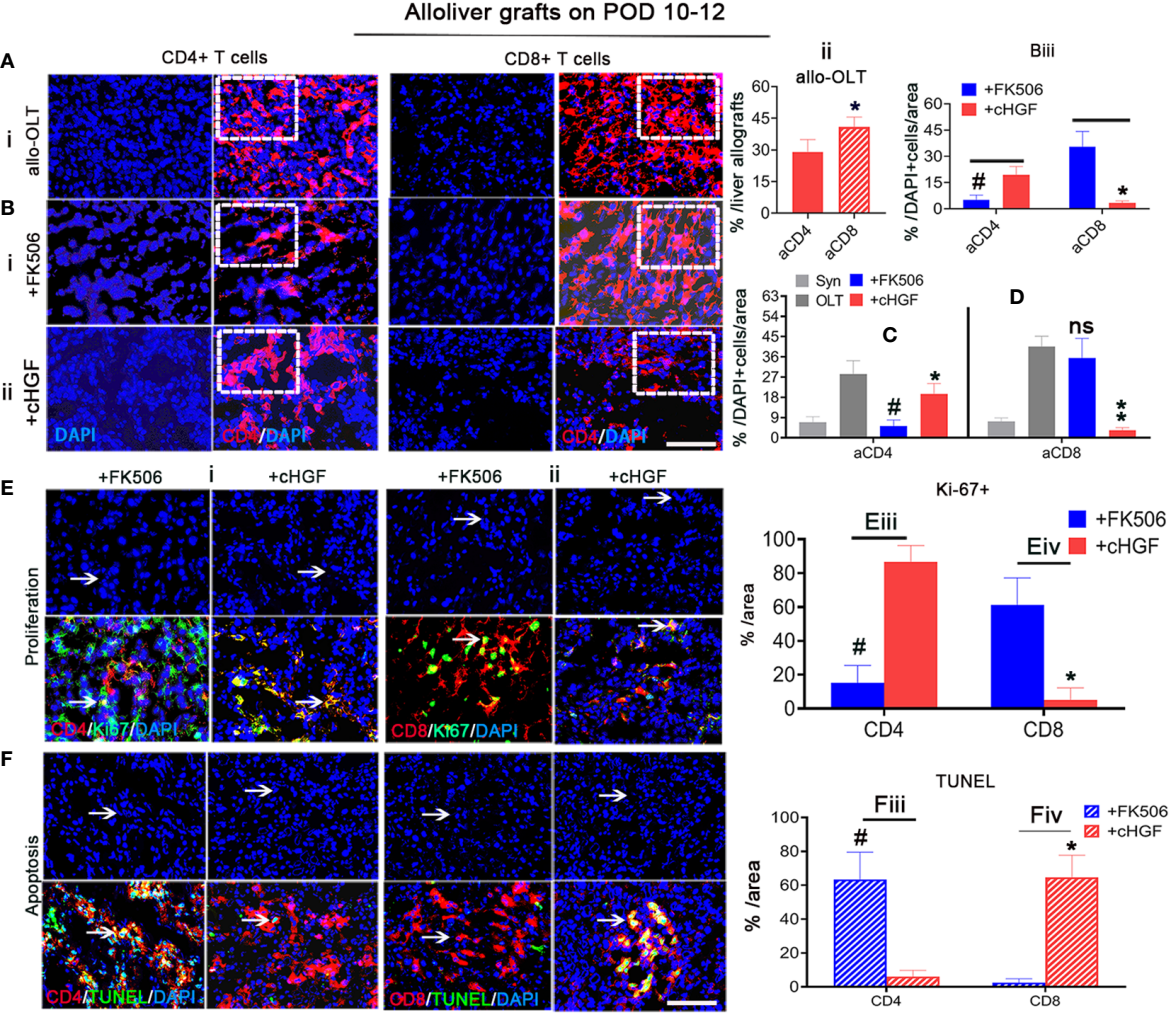
cHGF mainly suppressed the aCD8^+^ T cell subpopulation, while FK506 mainly affected the aCD4^+^ T cell subpopulation in liver allografts. **(A**i, ii**)** IF staining for CD4^+^ (red) and CD8^+^ (red, stripe) T cells (i, boxes) and quantitative analysis (ii). **(B**i–iii**)** IF staining for CD4^+^ (red) and CD8^+^ (red) T cells from the FK506 (i, boxes) and cHGF (ii, boxes) groups and comparative analysis of the proportions of CD4^+^and CD8^+^ T cells between the FK506 (blue bars) and cHGF (red bars) groups (iii, *n* = 6). **(C, D)** Analysis of aCD4^+^ T cells **(C)** and CD8^+^ T cells **(D)** in the Syn (gray bars) and allo-OLT recipient rats (black bars) treated with FK506 (blue bars) and cHGF (red bars); *n* = 6. **(Ei**–iv**)** Immunostaining (i, ii) and analysis for Ki-67 (green) within CD4^+^ (red, i, iii) and CD8^+^ (red, ii, iv) T cells (merged images, arrows) and their comparative analysis between the FK506 (blue bars) and cHGF (red bars) groups. **(Fi**–iv**)** TUNEL (green) staining of CD4^+^ (red, i, iii) and CD8^+^ (red, ii, iv) T cells (merged images, arrows) and comparative analysis of recipient liver allografts between the FK506 (blue-striped bars) and cHGF (red-striped bars) groups; the boxes represent a quarter of the whole stained field. Original magnification = ×400; scale bar = 200 µm. The data are presented as the mean ± SD of at least three independent experiments, *n* = 6. *#p < 0.05 vs*. CD4* and FK506; **p < 0.001 vs. the allo-OLT group; ns denotes no significance.

Given the more potent effect of cHGF than FK506 in decreasing the proportion of total CD3^+^ T cells, the FK506-mediated CD4^+^ T cells/IL-2 tolerance (43), and our previous evidence that HGF prolongs liver xenograft survival by promoting xenostimulated CD8^+^ T cell apoptosis (6), we speculated that allostimulated aCD4^+^ and aCD8^+^ T cell subpopulations may respond differently to cHGF and FK506. To assess this hypothesis, we immunostained liver allografts (**Figure 3**) and recipient immune organs (spleens) (**Supplementary Figure S2**). We first found that both aCD4^+^ (red) and aCD8^+^ (red) T cells were more strongly activated in the allo-OLT group (**Figure 3A**i; the boxes indicate a quarter of one field) on POD 10–12, but the proportion of aCD8^+^ T cells was shown to be significantly higher than that of aCD4^+^T cells (**Figure 3A**ii, a red striped bar); this confirmed that aCD8^+^ T cells are the main effectors in acute liver rejection (44). We next confirmed that the proportion of aCD4^+^ T cells was significantly decreased in the FK506 group (**Figure 3B**i, a box) compared with the cHGF group (**Figure 3B**ii, a box, iii, #*p* < 0.05), while aCD8^+^ T cells were more sensitive to cHGF than FK506 (Biii, *p < 0.05), suggesting that aCD4^+^ T cells and aCD8^+^ T cells in liver allografts are sensitive to FK506 and cHGF, respectively. Interestingly, compared with allo-OLT (**Figures 3C, D**, black bars) which had significantly increased CD4 and CD8 T cell subpopulations compared with the Syn group (**Figures 3C, D**, gray bars), cHGF (red bars) had a significant effect on the proportions of both aCD4^+^ (**Figure 3C**, red bar, *p < 0.05) and CD8^+^ T cells (**Figure 3D**, **p < 0.001), while FK506 (blue bars) affected only aCD4^+^ T cells (**Figure 3C**, #*p* < 0.05) and did not affect the proportion of aCD8^+^ T cells (**Figure 3D**, ns *p* > 0.05), suggesting that cHGF is able to decrease both CD4 and CD8 T cell subpopulations, while FK506 does so only with the CD4 T cell subpopulation during early tolerance induction. Similar biological phenomena were observed in recipient spleens (**Supplementary Figures S2A–D**). Moreover, double staining for Ki-67 and TUNEL showed a greater decrease in Ki-67^+^/CD4^+^ T cells (**Figure 3E**i, arrows, merged images) and an increase in the proportion of TUNEL^+^/CD4^+^ T cells (**Figure 3F**i, arrows, merged images) in the FK506-treated rats compared with the cHGF-treated rats (**Figures 3E**iii, Fiii, #*p* < 0.05). In contrast, a decreased proportion of Ki-67^+^ cells in the aCD8^+^ T cells (**Figure 3E**ii, arrows, merged images) and increased TUNEL expression (**Figure 3F**ii, arrows, merged images) were observed in the cHGF-treated rats compared with the FK506-treated rats (**Figures 3E**iv, **F**iv, *p < 0.05). Similar changes were observed in the recipient’s spleens (**Supplementary Figures S2E, F**), further verifying that aCD4^+^ T cells are hyporesponsive to FK506, while aCD8^+^ T cells are hyporesponsive to cHGF.

To determine whether h-rHGF treatment has a similar effect *in vitro* as cHGF has *in vivo*, we analyzed MACS-purified splenic CD4^+^ T cells and CD8^+^ T cells from allo-OLT model rats (on POD 7), and the FCM analysis showed that the purity of CD4^+^ and CD8^+^ T cells were more than 96% and 93%, respectively (**Supplementary Figure S3A**). These cells were stimulated with 0.5%–1% PHA (denoted as paCD4^+^ T cells and paCD8^+^ T cells) and cocultured with FK506 on paCD4^+^ T cells (**Supplementary Figure S3B**, 0.25–40 ng/mL) and h-rHGF on paCD8^+^ T cells (**Supplementary Figure S3C**, 50–400 ng/mL) for 24 h. A higher sensitivity of paCD4^+^ T cells to FK506 even at a very low dose was observed (**Supplementary Figure S3B**, 0.25 ng/mL, horizontal arrow, ###*p* < 0.0001), while paCD8^+^ T cells responded to h-rHGF in a dose-dependent manner; 50 ng/mL significantly increased the clonal numbers (**Supplementary Figure S3C**, #*p* < 0.05), while the clonal numbers significantly decreased at 200 ng/mL (**Supplementary Figure S3C**, *p < 0.05) and decreased even more obviously at 400 ng/mL (**Supplementary Figure S3C**, **p < 0.001). Furthermore, 1–8 ng/mL (equivalent to the optimal clinical dose) FK506 and 400 ng/mL h-rHGF were selected for further cocultures. After 24 h, we found that the expanded colonies formed different sizes (**Supplementary Figure S3D**) of small (white), middle (yellow), and large (pink), in which the colony number of paCD4^+^ T cells decreased significantly after treatment with FK506 (**Supplementary Figure S3D**—ii, left panel, iv, ###*p* < 0.0001), while almost no change was observed after treatment with h-rHGF **(** **Supplementary Figure S3D**—iii, left panel, iv, ns *p* > 0.05) compared with that after the PHA control treatment (**Supplementary Figure S3D**—i, left panel, iv, v, gray bars). In contrast, the colony number of paCD8^+^ T cells treated with h-rHGF decreased significantly (**Supplementary Figure S3D**—iii, right panel, v, **p > 0.001), but almost no changes were observed with the FK506 treatment (**Supplementary Figure S3D**—ii, right panel, v, ns *p* > 0.05) compared with the PHA control treatment, suggesting that FK506 and h-rHGF have different abilities to affect CD4^+^ and CD8^+^ T cell subpopulations: FK506 preferentially suppressed paCD4^+^ T cells, while h-rHGF preferentially suppressed paCD8^+^ T cells. These observations indicate that aCD4^+^ T cells are sensitive to both FK506 and cHGF but are more sensitive to FK506, while aCD8^+^ T cells are only sensitive to cHGF.

### Superior effects of FK506 over cHGF in modulating Tregs in the livers of allo-OLT model rats

**Figure 4:**
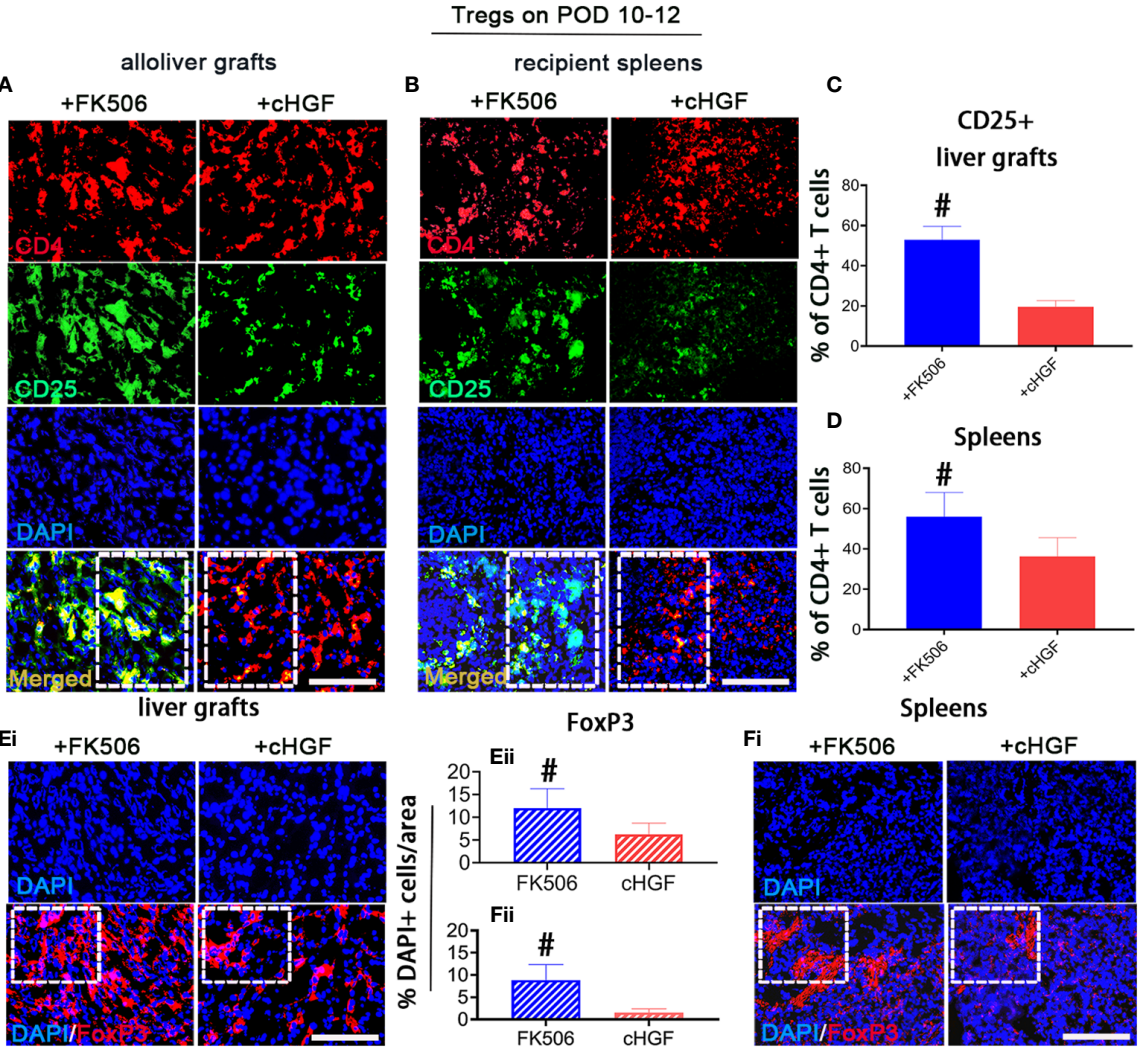
Superior effects of FK506 over cHGF in modulating Tregs in liver allografts. **(A–D)** Double IF staining for CD4 (red) and CD25 (green) (**A**, merged, boxes) and recipient spleens **(B)**, merged, boxes) after FK506 (blue bars) and cHGF (red bars) treatment and quantitative analysis of the data in **(A, C)**, *n* = 6) and **(B, D)**, *n* = 6); the boxes represent one-half area of the whole stained field. **(E**i–ii**)** Staining for FoxP3 (red) (i, boxes) after FK506 (blue-striped bars) and cHGF (red-striped bars) treatment and quantitative analysis (ii, *n* = 6/drug). **(F**i–ii**)** Staining for FoxP3 (red) in recipient spleens (i, boxes) after FK506 (blue-striped bars) and cHGF (red-striped bars) treatment and quantitative analysis (ii, *n* = 6/drug); the boxes represent a quarter acres of the whole stained field. Original magnification = ×400; scale bar = 200 µm. The data are presented as the mean ± SD of at least three independent experiments. *#p* < 0.05 vs. the cHGF group.

The Treg subpopulation is one of the key contributors to peripheral tolerance induction (45). Although cHGF and FK506 have both been reported to contribute to tolerance induction after LTx through these cells, we are the first group to compare their effects on Tregs after allogeneic LTx. Double IF staining for CD4/CD25, which are Treg markers (46), showed that the proportion of CD25^+^ (green) within CD4^+^ T cells (red) was significantly higher in both liver allografts (**Figure 4A**; the boxes indicate half of the field) and recipient spleens (**Figure 4B**, boxes) of the rats treated with FK506 (blue bars) compared with those of the allo-OLT model rats treated with cHGF (red bars) on POD 10–12 (**Figures 4C, D**, #*p* < 0.05), although both drugs promote the Treg population (not shown), suggesting that FK506 has a stronger effect than cHGF in regulating Tregs. Stained sections for FoxP3 (red), another Treg marker (47), in the same recipient tissues (**Figures 4E**i, **F**i, boxes) revealed a trend similar to the proportion of CD25^+^/CD4^+^ T cells. The number of FoxP3^+^ cells (red) was dramatically higher in the FK506 group (blue-striped bars) than in the cHGF group (red-striped bars) (**Figures 4E**ii, **F**ii, #*p* < 0.05), further confirming our findings. These observations indicate that, unlike cHGF, FK506 may induce allogeneic tolerance after LTx by affecting mainly the populations of both CD4^+^ T cells and Tregs.

### Superior effects of cHGF over FK506 in prolonging allograft survival and inducing functional recovery in the allo-OLT rat model

**Figure 5:**
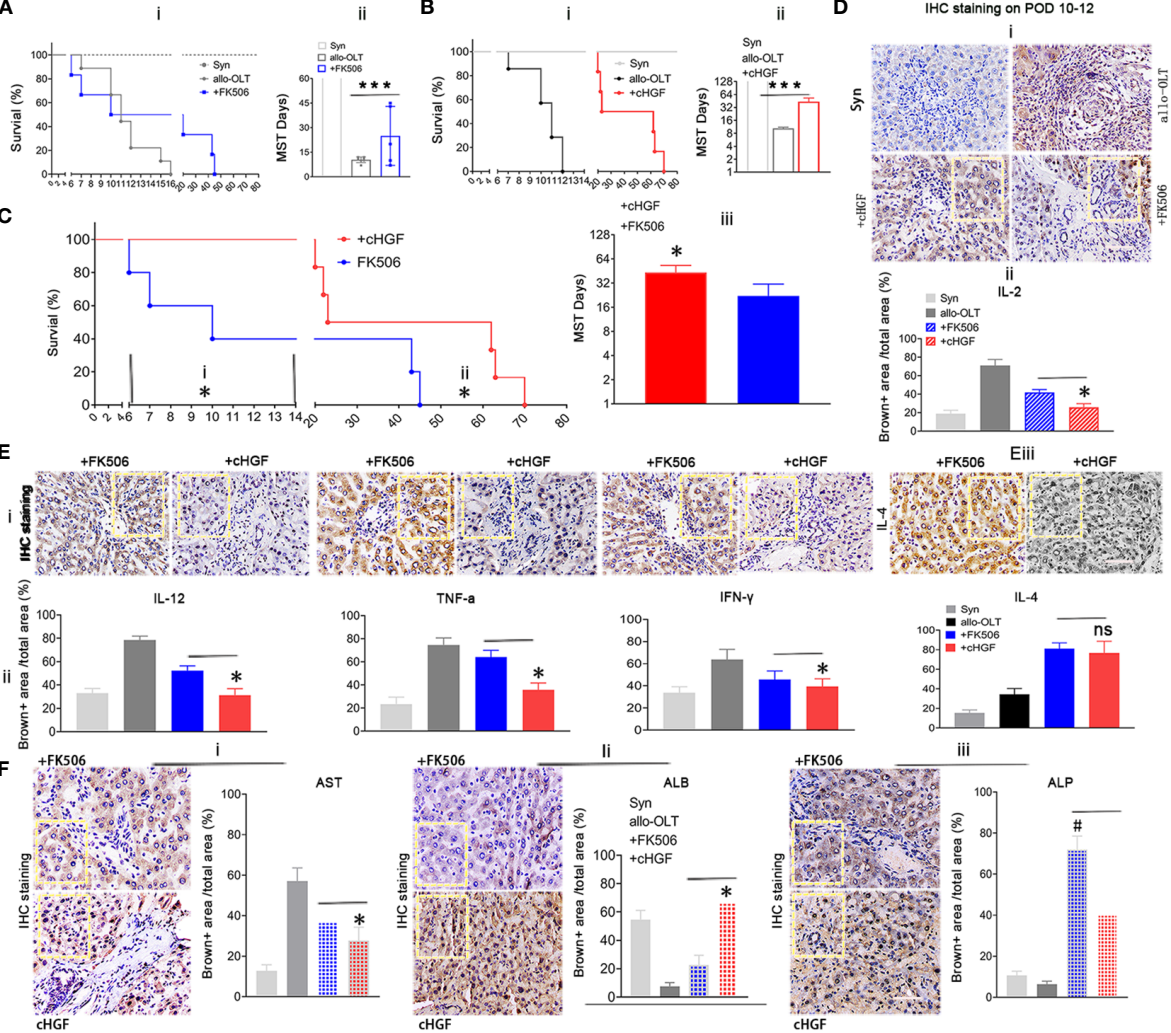
Superior effects of cHGF over FK506 in prolonging allograft survival and achieving functional recovery in the allo-OLT rat model. **(A**i, ii**)** Survival curve (i) and the MST/days (ii) in allo-OLT rat model (black, *n* = 24) following oral treatment with FK506 (blue, 0.2 mg/kg/day, equal to the clinical dosage, *n* = 24) and comparison of the model rats to Syn rats (gray, *n* = 10). **(B**i, ii**)** Survival curve of the rats that received cHGF (red, *n* = 24) compared with that of the allo-OLT (black, *n* = 24) and Syn rats (gray, *n* = 10) (i) and quantification of the MST/days (ii). **(C**i–iii**)** Allo-OLT model rats received FK506 (blue, *n* = 24) or cHGF (red, *n* = 24) in an early time period (i) and later time period (ii), and the later time period of MST was analyzed (iii). **(D**i, ii**)** IHC staining (brown) in subgroups (i, boxes) of IL-2 in liver allografts of the FK506 (blue-striped bar), cHGF groups (red-striped bar), the allo-OLT (black) and Syn (gray), and quantitative analysis (ii), *n* = 6. **(E**i–iii**)** The images of IHC staining (brown) for the proinflammatory cytokines IL-12, TNF-ɑ, IFN-γ (i, boxes), and the anti-inflammatory cytokine IL-4 (iii, boxes) in subgroups of liver allografts and comparative analysis (ii), with the analyzed data for the Syn (gray bars) and allo-OLT (black bars) groups (ii) from **Supplementary Figure S3E**—i, ii, boxes, *n* = 6). **(F)**i–iii**)** IHC staining for the functional hepatic proteins AST (i), ALB (ii), and ALP (iii) in liver allografts (boxes) of the different subgroups and quantitative analysis of those groups [Syn (gray); allo-OLT (black); cHGF (red latticed bar), and FK506 (blue latticed bar), *n* = 6). Staining for ALP and quantitative analysis showed that FK506 had a stronger effect on ALP levels (iii). The analysis also included the staining data for the Syn (gray bars) and allo-OLT (black bars) groups presented in **Supplementary Figure S3F**; *n* = 6; the boxes represent one-sixth of the whole stained field. Original magnification, ×400; scale bar =200 μm. The data are representative of at least three independent experiments and presented as the means ± SDs. *p < 0.05 vs. FK506; #*p *< 0.05 vs. cHGF, and ***p < 0.0001 vs. the allo-OLT group (Student’s *t*-test, two-way ANOVA); ns represents no significance.

Given the preferential effects of cHGF and FK506 on CD8^+^ T cells and Tregs, respectively, the key subpopulations in acute tolerance establishment, we investigated which has a more potent effect in inducing acute tolerance and functional improvement during acute phase after LTx. We injected (Mat/Met) cHGF (red) or FK506 (blue) into the allo-OLT recipient rats and found that graft survival was significantly (***P < 0.001) prolonged in both drug groups compared with the untreated allo-OLT group (**Figure 5A**i, ii, black), but the mean survival times (MST/days) prolonged significantly in the cHGF group (43.33 ± 23.91 days, *n* = 48, 24/time point) than in the FK506 group (20.22 ± 19.97 days, *n* = 48, 24/time point) compared with allo-OLT group (9.33± 3.24 days, *n* = 24, 12/time point) (“”Aii, Bii).

Looking over the prolonged graft survival in cHGF group than in the FK506 group compared the rats at early time points (before 2 weeks, before ~6–14 days, **Figure 5C**i), typical period of lethality days after LTx, and also at later time points (after 6 weeks, ~45–73 days (**Figure 5C**ii; the typical period of acute rejection time after LTx), a significantly longer MST was found in the cHGF-treated group (red) than in the FK506-treated group (blue) at both periods (**Figure 5C**iii, *p < 0.05), suggesting that cHGF has a stronger effect than FK506 on inducing early tolerance and maintaining the acute tolerance. In our study, the rats in the Syn group survived for more than 1 year, considered to be equivalent to indefinite survival after LTx (**Figures 5A, B**, gray, *n* = 20, 10/time point). IL-2 is commonly accepted to promote tolerance mechanisms by FK506 after organ transplantation (48); thus, we compared the effect on the allo-OLT rats after the two drug treatments. Using HIC staining (brown), we identified decreased IL-2 expression more obviously in the cHGF (red-striped bar) than the FK506 (a blue-striped bar) groups (**Figure 5D**i; the boxes indicate one-sixth area per field, ii, *p < 0.05), although both cHGF and FK506 decreased the IL-2 expression compared with that in the allo-OLT group (**Figures 5D**i, ii, black), which dramatically increased compared with that in the Syn group (**Figure 5D**i, ii, gray). Similar decreases in the expression of other proinflammatory cytokines, e.g., IL-12, TNF-α, and IFN-γ, and the anti-inflammatory cytokine IL-4 were observed in those subgroups (**Supplementary Figures S3E**—i, ii, **5E**—i–iii; the boxes indicate one-sixth area/field). Moreover, the hepatic proteins AST and ALB were observed by the same staining in the same subgroups (**Supplementary Figure S3F**, **5F**i, ii), such were consistent with histological changes (**Figures 2A, B**), suggesting the advantageous effects of cHGF over FK506 in improving functional recovery after LTx. Differently, in terms of IHC staining in these subgroups, hepatic ALP was stronger with FK506 (blue latticed bar) than with cHGF (red latticed bar) (**Figure 5F**iii, boxes, #*p* < 0.05), although both drugs improved this protein compared with the allo-OLT group (**Supplementary Figure S3F**, bottom panels, boxes; **Figure 5F**iii, black) which showed a lower expression than that of the Syn group (**Supplementary Figure S3F**, bottom panels; **Figure 5F**iii, gray), indicating that FK506 may be involved in bile duct improvement (49), while cHGF may be involved in hepatocytes. These observations suggest that the two drugs likely induce tolerance through different pathways: FK506 mainly affects ALP for bile duct recovery (49), while cHGF mainly affects ALB-mediated hepatocytes.

### cHGF induces aCD8^+^ T cell apoptosis through the HGF-only receptor cMet

**Figure 6:**
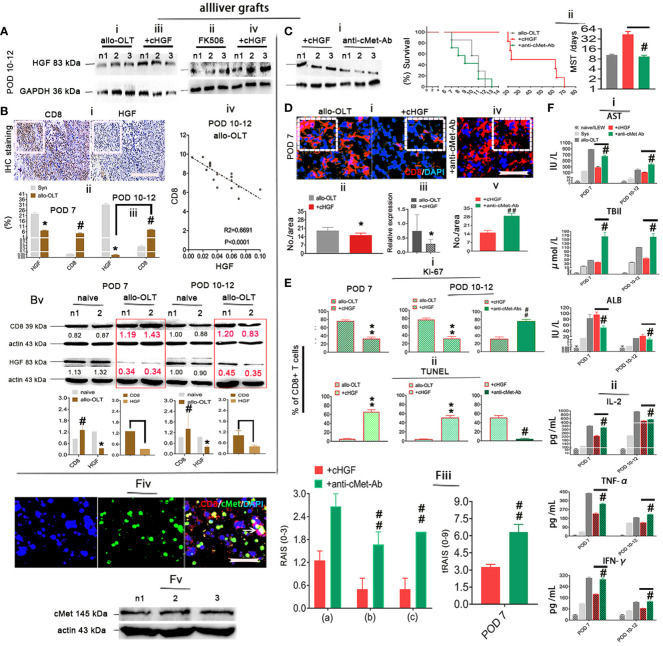
HGF induced aCD8^+^ T cell apoptosis in a mechanism mediated by its only receptor cMet. **(A**i–iv**)** Western blot analysis for HGF in liver allografts from recipient rats of the allo-OLT (i) that received FK506 (ii) and cHGF (iii iv) on POD 10–12; *n* = 6. **(B**i–v**)** IHC staining (brown) for HGF and CD8 in the same area of tissue specimens from the rats subjected to allo-OLT on POD 10–12 (i, boxes) (*n* = 6/group) and a comparison to the Syn groups on both POD 7 (ii) and 10–12 (iii); the correlations between CD8 and HGF in allo-OLT recipient rats on POD 10–12 (iv)*, n* = 25 points. Western blot analysis was used for the expression of HGF and CD8 in naïve livers (black) and livers from the rats subjected to allo-OLT (red in boxes) on POD 7 and 10–12 (Bv), densitometric quantification showing the decreasing trend in expression. The target protein expression was normalized to the expression of β-actin; n1 and n2 represent two individual samples; *n* = 3/time point. Original magnification ×200; scale bar = 100 µm. *#*p* < 0.05 vs. the Syn group. **(C**i, ii**)** Western blot detection for HGF expression in liver allografts from the recipient rats treated with cHGF plus anti-cMet-Ab (i, the same treatment with cHGF but without the IV injection approach) on POD 7 (*n* = 6) and survival curve with the MST analysis (ii). *n* = 16. #*p* < 0.05 vs. the cHGF group (Student’s *t*-test). **(D**i–v**)** IF staining for aCD8^+^ T cells (red) in the allo-OLT and cHGF groups at the protein (i, boxes, ii, *n* = 6) and mRNA (RT-qPCR, *n* = 3) (iii) levels on POD 7 and the cHGF plus anti-cMet-Ab group (iv, a box, v). **(E**i, ii**)** Analysis of the IF staining of Ki-67 (green) within CD8^+^ T cells (red) in liver allografts from the allo-OLT and cHGF groups on POD 7 and 10–12 shown in **Supplementary Figures S4D**—i, ii (i, **p < 0.001 vs. allo-OLT); analysis of TUNEL staining (green) within CD8^+^ T cells (red) in liver allografts from the allo-OLT and cHGF groups on POD 7 and 10–12 shown in **Supplementary Figures S4E**—i, ii (ii, **p < 0.001 vs. allo-OLT). Analysis of cHGF plus anti-cMet-Ab (solid blue bars) for CD8^+^ T cell proliferation (Ki-67) and apoptosis (TUNEL) in **Supplementary Figures S4D**—iii, iv, **E**iii, iv (i, ii, #*P* < 0.05 and #*p* < 0.05, ##*p* < 0.001 vs. the cHGF group), Student’s *t*-test, *n* = 6. Original magnification, ×400; scale bar = 200 µm. (**F**i–v) Analysis of the serum levels of AST, TBiL, and ALB (i); the cytokines IL-2, TNF-α, and IFN-γ (ii) in the naive (light gray bars), Syn (gray bars), allo-OLT (black bars), cHGF (red bars), and cHGF plus anti-cMet-Ab (green bars) subgroups by ELISAs; histological assessment by RAI score [RAISs (0–3) and tRAISs (0–9)] around PVs (a, boxes), BDs (b, boxes) and VEs (c, arrows) from **Supplementary Figures S4F**—i, ii on POD 7 and POD 10–12 according to Banff’s criteria (iii, #*p* < 0.05, ##*p* < 0.001 vs. the cHGF group, Student’s *t*-test), *n* = 10/time point, IF staining (*n* = 6) of cMet (green) in MACS-purified Lewis rat naive splenic CD8 T cells (red) (F—iv, the arrow denotes merged brown staining) and confirmation of cMet expression by Western blot analysis (**F**—v, *n* = 3, n1–n3 represent each set of individual cells); the boxes represent one-sixth of whole stained field. Original magnification, ×400; scale bar = 200 µm. The data are presented as the means ± SDs of at least three independent experiments. The asterisks indicate a statistically significant difference.

The main target of cHGF in other biological systems is HGF (12), and given our previous findings that HGF can induce the apoptosis of recipient xenogeneic CD8^+^ T cells (6), we speculate that cHGF may also exert its effects in the allo-OLT model through HGF. To test this hypothesis, we first assessed the expression level of HGF in liver allografts after cHGF administration. Western blotting revealed higher HGF levels in the allografts of the cHGF-treated recipients (**Figures 6A**iii, iv) than in those of both the OLT model and FK506-treated recipients (**Figures 6A**i, ii) on POD 10–12 (*n* = 3), suggesting that cHGF administration enhanced local endogenous HGF production. Then, we surmised that decreased HGF levels in allo-OLT rats may cause strong CD8^+^ T cell activity. IHC staining (**Figure 6B**i, boxes) in the same liver tissue fields of liver allografts confirmed that the expressions of HGF (on POD 10–12) (**Figure 6B**ii) were significantly lower and CD8 (on both POD7 and 10–12; the boxes indicate one-sixth area/field) was significantly higher (#*P* < 0.05) in the liver grafts from the allo-OLT recipient rats than in those from the syngeneic recipient rats (gray) (**Figures 6B**i–iii). An analysis of the immunostaining data from the allo-OLT recipient rats revealed that approximately 25%–40% of the CD8^+^ T cells were within DAPI^+^ cells (**Supplementary Figure S4A**, red bar) compared with the naive livers (**Supplementary Figure S4B**, less than 4% CD8^+^ T cells, red), and within the active aCD8^+^ T cells in liver grafts of allo-OLT model rats, less than 4% of these cells were targeted by TUNEL (**Supplementary Figure S4A**, green bar). Notably, we observed that the decrease in HGF expression and increase in CD8 expression on days 10–12 (**Figure 6B**iii) were significantly negatively correlated (*R* ^2 =^ 0.6691, **Figure 6B**iv, *P* < 0.0001). The results of the Western blot analysis of HGF and CD8 expression in liver allografts from the same animals revealed similar results (**Figure 6B**v, red; numbers in the red boxes), suggesting that the lack of HGF signals in allo-OLT rats suppresses aCD8^+^ T cell activity. In other systems, HGF signals through the tyrosine receptor kinase cMet, the best-characterized receptor for HGF (50). To determine whether cHGF/HGF signaling is also mediated by cMet, we treated allo-OLT model rats with a neutralizing antibody that blocks cMet function (anti-cMet-Ab) (2 × 200 ng/animal, intrasplenic, 2 days before and during grafting, mixed with cHGF treatment). HGF expression, which was assessed by Western blot analysis, was not increased (**Figure 6C**i, compared with **Figure 6A**i), and graft survival was also not prolonged in animals that received anti-cMet-Ab (green) (**Figure 6C**ii, *n* = 16/time point) compared with the allo-OLT rats (black, **Figure 6C**ii), while HGF (red) expression and graft survival were barely affected by FK506 (not shown). Immunostaining for CD8 (red) revealed suppressive effects by cHGF in the recipient’s grafts (**Figure 6D**i, boxes indicate a quarter area/field) both at the protein (**Figure 6D**ii) and mRNA (**Figure 6D**iii) levels and in the blood (**Supplementary Figures S4C**—i) on POD 7 after treatment were abolished by anti-cMet-Ab (**Figure 6D**iv, v, **Supplementary Figure S4C**—ii, solid green bars). Furthermore, double IF staining showed the decreased number of Ki-67^+^ green) and promotion of apoptosis (TUNEL^+^, green) (**Supplementary Figure S4D**—i) at POD 10–12 (**Supplementary Figures S4**—ii, **6E**i, left two charts) and promoted TUNEL expression (green) in grafts and recipient’s spleens on days 7 (**Supplementary Figures S4D**—i, **E**—i, boxes; **Figure 6E**i, left charts, green-striped bars) and 10–12 (**Supplementary Figures S4D**—ii, **E**—ii, boxes; **Figure 6E**ii, right charts, green-striped bars); these were also reversed by anti-cMet-Ab (**Supplementary Figures S4D, E**—iii, iv, boxes; **Figures 6E**i, ii, solid green-striped bar). These findings suggest that cHGF affects aCD8^+^ T cells both locally (in liver allografts) and in peripheral immune organs (the spleen) (**Supplementary Figure S5**) by inhibiting their division and stimulating their apoptosis via the blood in a mechanism dependent on HGF signaling. The ability of cHGF (red bars) to modulate the serum levels of AST, total bilirubin (TBil, a functional bile duct marker), and ALB (**Figure 6F**i), the cytokine profiles (**Figure 4F**ii), and the inflammatory infiltrative cells around the PVs [(a) boxes], BDs [(b) boxes], and VEs [(c) arrows] in grafted liver on POD 7 (**Supplementary Figure S4F**—i, top panels, boxes) and 10–12 (**Supplementary Figure 4F**—ii, top panels, boxes) stained by H&E and assessed by RAI scores (RAISs and tRAISs) in grafted liver (**Figure 6F**iii) was also abolished by treatment with anti-cMet-Ab (**Supplementary Figures S4F**, **6F**—ii, bottom panels, boxes, iii, solid green bars)—for example, significantly improved levels of AST, TBil, and ALB (*p < 0.05, **Figure 6E**i) and cytokines of IL-2, TNFα, and IFN-γ (**Figure 6E**ii, *p < 0.05) were decreased on POD 10–12 and had improved pathology on POD 7 (**Supplementary Figure S4F**—i, top panels) and 10–12 (**Figure 6E**ii, top panels) in cHGF (red bars)-treated animals that all can be reversed in animals that received anti-cMet-Ab (**Figures 6F**i–iii, #*p* < 0.05; ##*p* < 0.001 vs. cHGF). We detect cMet (green) to be expressed in MACS-purified aCD8^+^ T cells (red) using double IF staining (**Figure 6F**iv, arrow), and by using Western blotting (**Figure 6F**v), it was confirmed that the biological effects of cHGF in allo-OLT is mediated through cMet. These observations strongly suggest that cHGF/HGF/cMet signaling acts as a critical player in aCD8^+^ T cell-mediated acute tolerance induction by suppressing cMet-expressing aCD8^+^T cell activity, which leads to graft survival and functional recovery in allo-OLT model animals.

### Inhibition of Fas antagonism by cMet through cHGF/HGF in recipients with acute liver rejection triggers aCD8^+^ T cell FAS-mediated apoptosis

**Figure 7:**
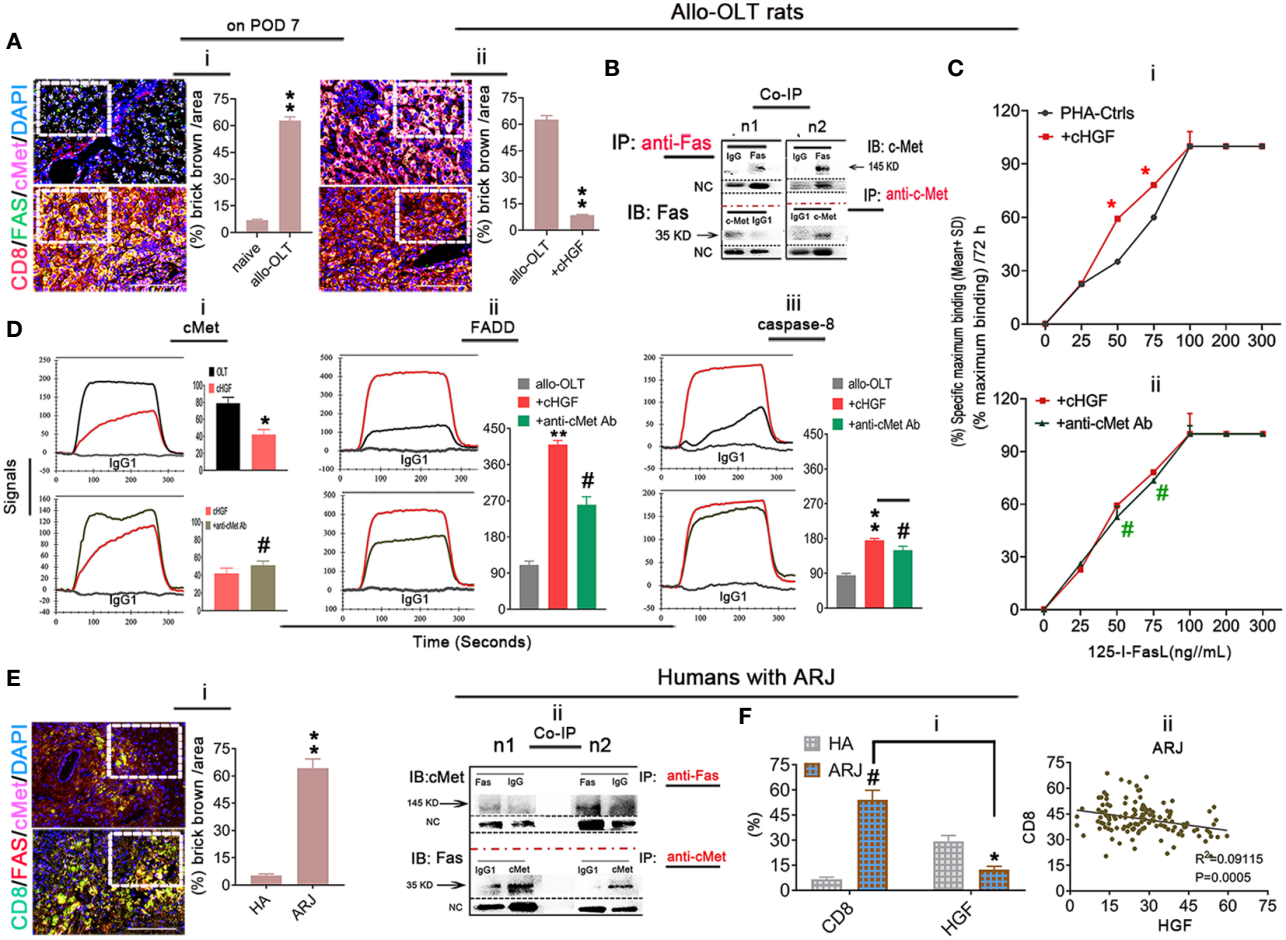
Inhibition of Fas antagonism by cMet through cHGF/HGF in recipients with acute liver rejection triggers aCD8^+^ T cell FAS-mediated apoptosis in allogeneic recipients. **(A**i, ii**)** WSIs of liver tissues taken with a multicolor automatic digital slide scanner and analysis (*n* = 6/group) of FAS (green) and cMet (pink) expression on aCD8^+^ T cells (red) in liver grafts from allo-OLT model rats on POD 7 (i, bottom box, merged signals are brick brown, shown in **Supplementary Figure S6A** with an arrow) and comparison of the brick brown-positive cells among total cells with naive livers (i, top box) and the rats (ii, top box) that received cHGF (ii, bottom box). **(B)** Co-IP was performed with either a Fas or cMet antibody followed by IB for cMet or Fas to assess the association between cMet and Fas on MACS-isolated Lewis rat naive splenic CD8^+^ T cells; n1 and n2 represent individual cells. **(C**i, ii**)** 125I labeling FAS-L showing FAS on CD8^+^ T cells formed aggregation with 400 ng/mL (higher levels) rHGF treatment for 72 h that had a significant higher affinity for 125I-labeled Fas-L (i, upper panel, *n* = 3), and anti-cMet-Ab blocked this interaction (ii, lower panel, *n* = 3). The data represent the average of three independent experiments with similar results. The red asterisks indicate a statistically significant difference. **(D**i–iii**)** Analysis of cMet (i), FADD (ii), and caspase-8 (iii) levels on splenic aCD8^+^ T cells after treatment with cHGF and anti-cMet-Ab by the LSPR method; *n* = 6/marker. **(E**i, ii**)** WSIs of human liver tissues with either ARJ (*n* = 6, i, bottom box) or HA (i, top box) taken with a multicolor automatic digital slide scanner and analysis of FAS (red) and cMet (pink) expression on CD8^+^ T cells (green) and HA liver tissues (*n* = 6, i, a top box) and analysis by Co-IP of the cMet-FAS interaction mode on human healthy peripheral MACS-purified CD8^+^ T cells (ii). **(F**i, ii**)** Analysis of the IHC staining (brown) data for CD8 and HGF in the same field of view of liver tissues from patients with ARJ (i, *n* = 6) presented in **Supplementary Figure S6D** (boxes, n1–n3) and HA liver tissues (*n* = 6) presented in **Supplementary Figure S6C** (boxes, n1–n3), indicate individuals and the correlation analysis between HGF and CD8 expression (ii) from patients with ARJ (**Supplementary Figure S6D**); the boxes represent a quarter area per stained field. The data are presented as the means ± SDs of at least three independent experiments. Original magnification, ×400; scale bar = 200 µm. *#*p* < 0.05 vs. the PHA-treated control or cHGF or HA group; **p < 0.001 vs. naive or allo-OLT or HA liver tissues (Student’s *t*-test, two-way ANOVA).

Given that the higher proportion of aCD8^+^ T cells is negatively correlated with the lower levels of HGF in allo-OLT recipient rats with acute liver rejection (**Figures 6B**iii–v) and our previous findings of HGF-mediated liver xenograft aCD8^+^ T cell apoptosis in xenogeneic liver transplantation rat model (6), we speculate that aCD8^+^ T cells exhibit elevated apoptosis in allogeneic recipients due to the crosstalk of HGF (from cHGF) to the death receptor FAS, resulting in FAS–mediated apoptosis and establishing tolerance, which can rapidly prevent graft rejection. Using a WSI automatic image scanner, we found Fas (green) antagonism by cMet (pink) in mouse liver allograft aCD8^+^ T cells (red), as shown by the merged brick color (**Figure 7A**i, bottom panel, box; **Supplementary Figure 6A**, indicated by an arrow) which was stronger in the allo-liver grafts on POD 7 than that from naive ones (**Figure 7A**i, top panel, box, **p < 0.05), and the merged color changed to a lighter shade from the OLT group (**Figure 7A**ii, top panel, box) to the cHGF-treated group (**Figure 7A**ii, bottom panel; the boxes indicate a quarter of one field, **p < 0.001). When analyzing the relationship of two molecules using Co-IP, we, for the first time, find the association of cMet and FAS with MACS-purified naive splenic mouse CD8^+^ T cells (**Figure 7B**) (51). These findings may suggest that FAS antagonism by cMet on aCD8^+^ T cells correlates with aCD8^+^ T cells/higher activity/tolerance failure/rejection; we deduced that HGF/cHGF activated cMet through its ligand HGF from cHGF, parting the association, FAS-free and aggregation, and then undergo FAS-mediated apoptosis. Using ^125^I labeling FasL (^125^I labeling FasL) (Mat/Met), we deduced FAS assembly in sensitive response to Fas ligand (FASL) after HGF administration of 400 ng/mL h-rHGF significantly increased aCD8^+^ T cell binding to ^125^I-FasL at 50 and 75 ng/mL during 72-h cultures (**Figure 7C**i, red line, *p < 0.05), and this effect was blocked by anti-cMet-Ab (green line, **Figure 7C**ii, #*p* < 0.05**)**, suggesting that cMet acts as a potent FasL antagonist and that its ligand HGF is a promoter of Fas trimerization (**Supplementary Figures S4E**, **S5C**, arrows indicate FAS trimerization). Additionally, by performing LSPR spectroscopy of metallic nanoparticles (*Mat/Met*), we showed in cultures that h-rHGF (red) significantly decreased cMet expression on MACS-purified splenic aCD8^+^ T cells (**Figure 7D**i, top panel) and promoted death-inducing signaling complex (DISC) formation, hallmarks of FAS-mediated apoptosis (52) through increased recruitment of FADD (**Figure 7D**ii, top panel) and caspase-8 (**Figure 7D**iii, top panel) to Fas within 72 h, compared with the cells from allo-OLT rats (**Figures 7D**i–iii, top panel, black). We used IgG1 (**Figures 7D**i–iii, gray lines**)** as controls. The effects of h-rHGF were inhibited by anti-cMet-Ab (**Figures 7D**i–iii, bottom panels, green), suggesting that the effects of HGF (in the form of cHGF) in recipient rats triggered FAS-mediated aCD8^+^ T cell apoptosis through cMet. Interestingly, we found that the data obtained in animals are largely similar to those obtained in humans. We were fortunate to obtain 12 human recipient liver sections (~1 cm^3^) from each individual with acute liver rejection (denoted as ARJ). The ARJ was diagnosed by two professional pathologists based on Banff’s criteria, and H&E staining of infiltrating cells around the PVs [(a) boxes], BDs [(b) boxes] and VEs [(c) arrows] was performed (**Supplementary Figure S6B**—ii) and assessed by RAI scores of RAISs and tRAISs (**Supplementary Figures S6B**—iii, iv). We used distal noninflamed hemangioma liver tissues as controls (denoted as HA here, *n* = 6, **Supplementary Figures S6B**—i, iii, iv; n1–n3 denote individual samples). The WSI automatic image scanner detected similar expression patterns of Fas (red) and cMet (pink) in human allostimulated CD8^+^ T cells (h-aCD8^+^ T cells, green) (**Figure 7E**i; the boxes indicate a quarter of one field) to those in the rats (**Figure 7A**i). Co-IP assays revealed that cMet and FAS interacted in healthy h-aCD8^+^ T cells isolated from peripheral blood (**Figure 7E**ii, n1–n2 denote individuals). Opposite expression patterns of CD8 and HGF were observed in ARJ livers (**Supplementary Figure S6D**) and HA tissues (**Supplementary Figure S6C**; the boxes indicate one-sixth area per field) at the same areas, identified by IHC staining (**Figure 7F**i). A similar linear correlation to the allo-OLT rat model (**Figure 6B**iv) was observed between CD8 and HGF expression in the ARJ human liver allograft samples (**Figure 7F**ii, *R* ^2 =^ 0.09115, *p* = 0.0005), suggesting that the resistance of h-aCD8^+^ T cells (green to apoptosis; TUNEL, red) in ARJ (**Supplementary Figure S6E**, merged, denoted as an arrow in the images and a chart; n1–n4 denote individuals) is also due to the lack of cMet activation by its ligand HGF. These data indicate that activation of cMet by its ligand HGF may allow aCD8^+^ T cells to cross immune barriers for acute tolerance induction in recipients with acute liver rejection.

### cHGF protects against FK506-mediated nephrotoxicity in allo-OLT model rats through HGF via an IL-10-related mechanism

**Figure 8:**
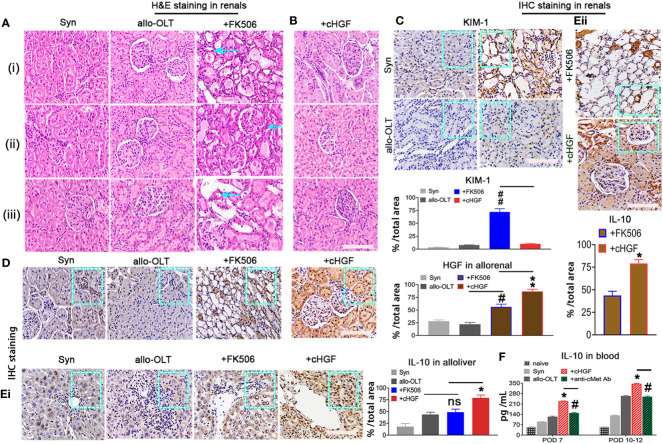
cHGF protects against FK506-mediated nephrotoxicity in the allo-OLT rat model *via* an IL-10-related mechanism. **(A)** H&E staining to evaluate the CNIT score in renal tissues (**Table 2**) from the Syn, allo-OLT, and FK506 (scores of 2, 2, and 1) subgroups based on the histologic features shown in **Table 2**, specifically proximal tubules with cytoplasmic isometric vacuoles or tv [(i) a blue arrow], tubular atrophy with a striped pattern of interstitial fibrosis [(ii) a blue arrow], and subendothelial, medial, and peripheral arteriolar hyalinosis or ah [(iii) a blue arrow]; *n* = 6/subgroup. **(B)** Identical analysis in the cHGF group based on CNIT scores shown in **Table 2** as 0; *n* = 6. **(C)** IHC staining for KIM-1 in renal tissues from the four subgroups and subsequent analysis showing a higher expression of KIM-1 in the FK-506 group (blue box and bar) than cHGF (blue box, red bar), other [Syn (gray bar), and allo-OLT (black bar)] groups (ii); *n* = 6/per subgroup. **(D)** IHC staining for HGF in renal tissues from the same subgroups and analysis of the different HGF expressions among the groups, *n* = 6/group. **(E**i, ii**)** IHC staining and analysis for IL-10 in the four subgroups of liver allografts (i) and in the two subgroups of FK506 and cHGF of renal tissues (ii) on POD 7; the boxes represent one-sixth area of the whole stained field. **(F)** ELISAs for serum IL-10 in subgroups (*n* = 6), including the naive (light gray bars), Syn (gray bars), allo-OLT (black bars), cHGF (red striped bars) and cHGF plus anti-cMet-Ab (solid green striped bars) on POD 7 and 10–12. Original magnification = ×400; scale bar = 200 µm. The data are presented as the mean ± SD of at least three independent experiments. *#p < 0.05 vs. the FK506 or allo-OLT group; **##p < 0.001 vs. the FK506 or cHGF group; ns indicates not significant, *p* > 0.05.

FK506 treatment after LTx commonly causes nephrotoxicity in the clinic (approximately 15%–20% of patients) (53), and here we show that cHGF can protect against FK506-mediated nephrotoxicity in allo-OLT model rats through HGF production. Nephrotoxicity was evaluated based on CNIT scores (54) via H&E staining (*Mat/Met*). FK506 indeed caused a significant injury to renal tissues on POD 10–12, which appeared as tubular isometric vacuolization (tv, i), striped interstitial fibrosis (ii), and peripheral or medial (ah, iii) as identified (**Figure 8A**, blue arrows; **Table 2**, blue checks markers, CNIT scores = 2(i), 2(ii), and 1(iii) respectively). The scores were higher than those in the Syn and allo-OLT subgroups (**Figure 8A**; **Table 2**, black check markers, CNIT scores = 0). The animals that received cHGF did not show any injury (**Figure 8B**; **Table 2**, red check markers, CNIT scores = 0**)**. IHC staining (brown; the boxes indicate one-sixth area/field) for TIM-1, a marker of kidney injury (55), revealed strong upregulation in the FK506 group (blue bar) (**Figure 8C**, top right panel, ##*p* < 0.001) but not the cHGF(red bar)-treated group (**Figure 8C**, bottom right panel) red bar) which was similar to the Syn (top left panel, gray bar) and allo-OLT(bottom left panel, black bar) subgroups (**Figure 8C**). These results further confirmed that cHGF alleviated injury. Interestingly, given that anti-cMet-Ab inhibited cHGF effect in blood (**Supplementary Figure S4C**—ii), also in liver allografts (**Figures 6A**iii, iv) than FK506 (**Figure 6A**ii), we investigated whether HGF expression also increases in renal tissues after cHGF treatment compared with FK506 treatment. Using the same IHC staining in subgroups, we confirmed the hypothesis and found a higher expression of HGF in renal tissues of the cHGF-treated group than in the FK506-treated group (**Figure 8D**, boxes, **p < 0.001), although HGF expression was also increased in the FK506-treated group which was higher than the Syn (gray bar) and allo-OLT (black bar) subgroups (**Figure 8D**, #*p* < 0.05). Notably, although the staining in liver allografts detected dramatically increased the IL-10 expression in the cHGF-treated group over the allo-OLT group (**Figure 8E**i; the boxes indicate one-sixth area per field, *p < 0.05), this did not occur in the FK506-treated group (blue bar, **Figure 8E**i, ns *p* > 0.05). Using the same staining further detects the expression of IL-10 in renal tissues; we found a significantly higher level of IL-10 expression in the cHGF-treated rats than in the FK-506-treated rats (**Figure 8Eii**). The ELISA assay confirmed that IL-10 expression increased in the blood of the recipient rats treated with cHGF (red-striped bars) compared with the allo-OLT recipient rats (black) on both POD 7 and 10–12 (**Figure 8F**, *p < 0.05), and these effects were abolished by anti-cMet-Ab (**Figure 8F**, green-striped bars, #*p* < 0.05). These findings suggest that HGF, produced in response to cHGF, may systematically and locally promote IL-10 production to help protect against renal injury via cMet (HGF dependent). These observations indicate that the superior effect of cHGF to FK506 in protecting against nephrotoxicity is due to the influence of HGF signaling on IL-10 activation not only in liver allografts but also in the kidneys through the blood.

Taken together, these studies suggest that the superior ability of cHGF to induce acute tolerance in the allo-OLT rat model relative to that of FK506 is due to its ability to stimulate HGF/cMet signaling, resulting in the activation of cMet-expressing aCD8^+^ T cell apoptosis, enhancement of functional recovery, and promotion of IL-10 production to protect the kidneys.

## Discussion

LTx is a life-saving procedure for ESLD of various etiologies, and both central and peripheral tolerance have been studied in an effort to achieve acceptance of allograft tissues in liver transplant recipients (56). Early on, high rates of acute graft rejection were overcome by the introduction of potent IS regimens, which are still largely in use today. However, long-term improvements in LTx procedures and patient outcomes have been hampered by the cumulative toxicity of maintenance IS therapy (57). FK506, for instance, was confirmed to be an IS in early studies and is currently the most commonly used drug in the clinic for acute tolerance induction after LTx (58); however, it can cause infection, nephrotoxicity, and incomplete preservation of graft function during tolerance induction (59). Therefore, novel immunosuppressive strategies for active liver tolerance induction and long-term good prognosis are urgently needed. In the present study, we extensively investigated the effects of FK506 therapy on acute tolerance induction, functional recovery, and renal injury protection by comparing them with the effects of HGF from a commonly clinically used HGF-promoting drug (cHGF) in a rat allo-OLT model to assess the clinical relevance of HGF as a novel tolerance inducer.

HGF-induced tolerance in organ transplantation has been reported (6). In the present study, we showed that the advantages of cHGF monotherapy over FK506 in beneficial effects are sustained in the allo-OLT rat model through HGF signaling. We have previously reported that 10–12 days of HGF/CD8^+^ T cell-mediated stem cell treatment facilitates the induction of xenogeneic tolerance for liver xenograft survival in a full MHC mismatch hamster-to-Lewis LTx rat model (MST, only 3–5 days) (6), suggesting the possibility of tolerance effect of HGF in allogeneic LTx. As expected, here using a well-established allo-OLT rat model based on Kamada’s two-cuff technique, we showed that the MST in the cHGF/HGF-treated recipient rats was much longer (MST: 43.33 ± 23.91 days) than the rats treated with FK506 (MST: 20.22 ± 23.91 days), although FK506’s MST was significantly longer than that of the untreated allo-OLT controls (MST: 9.45 ± 23.91 days). The 40-day liver allograft survival rate was approximately 21.81% in the rats treated with cHGF/HGF and 0% in the rats treated with FK506 alone, consistent with the results reported by Yamada et al. (60).

However, the mechanism underlying these differences is currently unclear. We show in the present study that the difference is due to T cell subpopulations: the action of HGF from cHGF is against the recipient aCD8^+^ T cells (6), which are the main mediators of graft acute rejection (61), and the effect of FK506 is on aCD4^+^ T cells and Tregs in recipients with LTx (6). The observations of the present studies showed that cHGF exerts a stronger effect on liver allograft survival in rats than FK506 mainly through the HGF-mediated suppression of proliferation (**Supplementary Figures S4D**, **S5B**, **6E**i) and promotion of apoptosis (**Supplementary Figures S4E**, **S5C**, **6E**ii) in recipient aCD8^+^ T cells by binding the HGF-only receptor cMet on these cells (**Figure 6F**), disturbing the spatial segregation of FAS by cMet (HGF-only receptor) (**Figures 7B, E**ii) (62, 63), which caused FAS assembly (**Supplementary Figure S4E**), arrows that indicate sensitivity to FAS-L (**Figure 7C**i), and inducing these cells to undergo FAS-mediated apoptosis (**Figure 7D**). These results indicate that HGF is able to cross the aCD8^+^ T cell immune barrier for acute tolerance induction and leads to liver allograft survival, consistent with our previous studies in a xenogeneic rat model (6), while our observations show that FK506 prefer to suppress recipient aCD4^+^ T cells and cMet is not be expressed on the cells (64), indicating that FK506 may not be able to cross the aCD8^+^ T cell barrier.

Furthermore, we investigated whether the mechanism(s) of proliferation and apoptosis of aCD8^+^ T cells is directly induced by HGF. A dose-dependent response of the recipient aCD8^+^ T cells to h-rHGF in cocultures suggests that the double-edged sword effect (pro-/antiapoptosis) of HGF (65–68) is also reflected in our allo-OLT recipient rats, and lower levels of HGF (<50 ng/mL)-induced CD8^+^ T cell DNA synthesis were not effective in dissociating cMet from Fas (**Figures 7A**i, **E**i), indicating that cMet and FAS preexist as a complex on CD8^+^ T cells from both rats (**Figure 7B**) and humans (**Figure 7E**ii); higher concentrations of HGF (>200 ng/mL) may disturb the antagonist effect of cMet (62) on FAS, causing FAS to dissociate from cMet and aggregation (**Supplementary Figures S4E**, **S5C**, arrows), enhancing sensitivity to FasL binding (**Figure 7C**i), recruiting FADD and caspase-8 (**Figures 7D**ii, iii), and eventually inducing Fas-mediated apoptosis (62), indicating that a lack of cMet activation by its ligand HGF leads to aggressive aCD8^+^ T cell activity in recipients with acute liver rejection (**Figures 6B**, **7F**).

Moreover, our clinical data showed for the first time that FK506 significantly suppressed lymphocyte proliferation at an early time point (3 weeks after FK506 administration), but which lymphocyte subpopulations are involved in these recipients remains unclear. We show herein the effect by FK506 preferentially on the CD4^+^ T cell subpopulation both *in vivo* and *in vitro*. However, the mechanism of aCD4^+^ T cell-mediated anti-rejection in allo-recipients is still unclear. FK506 may act on Tregs, either CD4^+^ Tregs (69, 70) or CD8^+^CD45RC^-^ Tregs (71), rather than directly acting on aCD8^+^ T cells. The finding in the present study that FK506 has a more potent effect in regulating CD4^+^/CD25^+^/FoxP3^+^ Tregs than cHGF/HGF (**Figure 4**) may explain the mechanisms, although HGF has also been suggested to play a role in tolerance in Treg regulation after LTx (6, 7, 63, 72).

To our knowledge, this study provides the first evidence that HGF (in the form of cHGF) and FK506 differ in their preferential targets in various T cell subpopulations in allogeneic recipients after LTx. The finding of cHGF/HGF-mediated environmental factor-related functional recovery in allo-OLT recipient rats is consistent with the data that recipients release a variety of cytokines and haptic proteins and show an improved pathological load. Under multiple conditions, the biological effects of FK506 on tolerance induction for graft survival after LTx occurs through inhibiting the IL-2 mechanism (73, 74). In this study, we showed that HGF resulted in an even more obvious inhibition of cytokine. In addition, IL-10 production could be another interesting issue to interpret the difference. We showed that cHGF/HGF treatment enhanced IL-10 production in liver allografts, consistent with previous studies using h-rHGF treatments in organ transplantation animal model (10, 11), which did not occur with FK506 treatment. Additionally, we confirmed a protective role of IL-10 against FK506-mediated renal toxicity (75), which is related to HGF production after cHGF monotherapy, inconsistent with the results reported by Ido et al. (76), indicating as HGF/IL-10 a defense mechanism for FK506-mediated renal toxicity in recipients with LTx. Clinically, the investigated phenomena in allo-OLT animals are consistent with those in humans with ARJ, indicating clinically related differences that activate cMet signaling with its ligand HGF agonists to target recipient aCD8^+^ T cells. Meanwhile, protection of FK-506-mediated renal injury or regulating Tregs with FK506 or both to target recipient aCD4^+^ T cells could have a value for acute tolerance induction after LTx.

In conclusion, FK506 is currently a commonly used IS in the clinic after LTx, but its effects are not ideal—that is, this drug does not induce appropriate liver allograft tolerance while avoiding general immunosuppression and nonimmunologic adverse effects. Our data demonstrated the advantages of HGF over FK506 in acute tolerance induction after LTx. Induction of recipient aCD8^+^ T cell apoptosis via activation of cMet on these cells for acute tolerance induction by its ligand HGF also protected against FK506-mediated renal injury. Thus, HGF may become a novel tolerance inducer for strategies of acute tolerance induction after LTx. Further research will likely focus on approaches for stimulating HGF signaling to induce this phenomenon. As HGF has a short half-life *in vivo* (77), new methods for delivering HGF need to be considered. Additional studies aimed at increasing the effective duration of HGF and improving the bioavailability of HGF using antibodies or agonists will help to further elucidate the role of HGF as a potential tolerogenic agent. Finally, we propose that HGF may play a role in LTx either in combination with FK506 application or as a monotherapy in further clinical studies.

## Data availability statement

The original contributions presented in the study are included in the article/**Supplementary Material**. Further inquiries can be directed to the corresponding authors.

## Ethics statement

The studies involving human participants were reviewed and approved by Human Tissue Bank (humans; registration number: Ky2022114) of Army Medical University. The patients/participants provided their written informed consent to participate in this study. The animal study was reviewed and approved by SYXK YU 2012–0012. Written informed consent was obtained from the individual(s) for the publication of any potentially identifiable images or data included in this article.

## Author contributions

Conceptualization and study design: HZ and LB; methodology: QC, ZY, HL, and LB; data curation and formal analysis: QC, JL, DH, MY, ZW, WL and LB; resources: QC, JL, ZP, and ZL; investigation: QC, YW, YH, JL, DH, MY, SL, and ZS; visualization: QC, WY, YC, ZP, ZL, and LB; funding acquisition: HZ, LZ, and LB; project administration: JL, ZL, and LB; supervision: LB; writing—original draft: HZ; writing—review, editing, and proofreading the submitted manuscript: LB. All authors contributed to the article and approved the submitted version.

## Supplementary material

Supplementary Figure 1 Surgery for establishment of the DA rat-to-Lewis rat allogeneic orthotopic liver transplantation model (allo-OLT). **(A)** Donor liver preparation. Donor livers were removed from DA rats, flushed with 15 mL of cold saline solution (40 units/mL) and stored at 4°C until transplantation. **(B—i–iv)** Recipient liver preparation (vascular denudation). The PVs (i, arrow) and pyloric veins (ii, arrow) were isolated and ligated with an 8–0 suture. The right suprarenal vein (iii, arrow) was severed between the 8–0 sutures, and the liver was freed from its posterior ligaments by cutting under gentle traction. The IVC (iv, arrow) was isolated down to the left renal vein. **(C—i–v)** Transplantation of donor’s liver into the recipients (vascular anastomosis). The donor’s liver was placed into the abdominal cavity, and end-to-end anastomosis between the suprahepatic vena cavas (SVCs) of the graft and the recipient (i, box) was performed. The recipient’s PV was flushed with UW solution, and a cuff was inserted into the vessel. An 8–0 suture was then secured to the recipient’s PV around the cuff (ii, box). The clamps on the SVC and the PV were removed to reestablish flow. Finally, the IVC (iii, box) and BD cuffs (v, box) (iii–v) were connected with an 8–0 suture. (**D**) H&E staining of infiltrating cells around the PVs (a, boxes), BDs (b, boxes), and VEs (c, arrows) in the liver allografts of Syn rats on POD 7, *n* = 6. **(E—i–iv)** The same stained liver allograft serial 5-μm-thick sections (i, iii) and scored RAISs on POD 7 (ii) and 10–12 (iv), *n* = 6/group/time point. **(F)** The same staining in liver tissues from rats subjected to allo-OLT on POD 7 and 10–12 (boxes) and assessment of tRAISs from sum of RAISs, *n* = 6/group/time point. Original magnification = ×200; scale bar = 100 µm. The data are presented as the mean ± SD of at least three independent experiments. **p* < 0.05, ***p* < 0.001 vs. the Syn group either on POD 7 or 10–12.

Supplementary Figure 2 cHGF mainly suppressed the aCD8^+^ T cell subpopulation, while FK506 mainly affected the aCD4^+^ T cell subpopulation in the recipient’s spleens. **(A—i, ii)** IF staining (i, boxes) for splenic aCD4^+^ (red) and aCD8^+^ (stripe) T cells in the allo-OLT group (i, boxes) and quantitative analysis (ii). **(B—i–iii)** The same staining for aCD4^+^ (red) and aCD8^+^ (red) T cells of the rats that received FK506 (i, boxes) and cHGF (ii, boxes) and comparative analysis of the proportions of CD4^+^ and CD8^+^ T cells between the FK506 (blue bar) and cHGF (red-striped bar) groups (iii); *n* = 6. **(C, D)** Analysis of recipient aCD4^+^ T cells **(C)** and aCD8^+^ T cells **(D)** in the Syn (gray) and allo-OLT (gray stripe) groups following FK506 (blue) and cHGF (red stripe) treatment; *n* = 6/group; the boxes represent a quarter area of the whole stained field. **(E—i–iv)** Double IF staining for Ki-67 (green) (arrows) within aCD4^+^ (red, i, iii) and CD8^+^ T cells (red, ii, iv) and comparative analysis of the positive cell numbers in the T cell subpopulations from the tissue sections of the rats treated with FK506 (blue bars) and cHGF (red striped bars). **(F—i–iv)** TUNEL staining (arrows) of aCD4^+^ (i, iii) and aCD8^+^ (ii, iv) T cells and comparative analysis of the same tissue sections treated with FK506 (blue bars) and cHGF (red-striped bars). Original magnification = ×400; scale bar = 200 µm. The data are presented as the mean ± SD of at least three independent experiments. **p* < 0.05 vs. either the allo-OLT or FK506 group*; *#p* < 0.05 vs. the CD4^+^T cells or FK506 or cHGF group; *##p* < 0.001 vs. the allo-OLT group; ns *p* > 0.05.

Supplementary Figure 3 Treatment of recipient aCD4^+^ and aCD8^+^ T cells with FK506 and h-rHGF in cocultures and analysis of cytokines and hepatic proteins in liver allografts by IHC staining. **(A)** FCM analysis of MACS-purified splenic aCD4^+^ T cells and aCD8^+^ T cells; *n* = 3. **(B)** A total of 1 × 10^5^ of the CD4^+^ T cells were treated with FK506 at 0.25–40 ng/mL for 24 h; all concentrations resulted in these cells’ suppression compared with PHA-stimulated aCD4^+^ T cells (as controls), and 1–8 ng/mL (blue line) was selected for the coculture studies. **(C)** The same number of aCD8^+^ T cells as aCD4^+^ T cells were treated with h-rHGF at different concentrations for 24 h, and ≤50 ng/mL h-rHGF significantly promoted sphere growth (an image represents more spheres vs. PHA-Ctrl, an image); ≥200 ng/mL, h-rHGF greatly (**p* < 0.05) decreased the number of spheres compared with the PHA control; at 400 ng/mL, the results were even more significant (***p* < 0.001, an image) compared with the PHA-Ctrl; thus, 400 ng/mL h-rHGF was selected for the *in vitro* clonal expansion study. **(D—i–v)** 0.5-1% PHA stimulated the CD4^+^ and CD8^+^ T cells (i) treated with FK506 (1–8 ng/mL) (ii) or h-rHGF (iii) for 24 h in cultures; then, expanded clonal numbers were counted from small, middle, to large as arrows of pink, yellow, and white, respectively, and quantitative analysis was separately performed for CD4 (iv, FK506: blue bars) and CD8 (v, h-rHGF: red bars); the box represents one-half area of the whole stained field. **(E—i, ii)** IHC staining for the proinflammatory cytokines IL-12, TNF-α, and IFN-γ (i, boxes) and anti-inflammatory cytokine IL-4 (ii, boxes) and their quantification analysis in shown as gray and black bars. **(F)** IHC staining of liver allograft sections for the hepatic proteins AST, ALB, and ALP from the Syn and allo-OLT groups (boxes); *n* = 6/protein/cytokine, and their quantification analysis is shown in as gray and black bars; the boxes represent one-sixth area of the whole stained field. Original magnification = ×200; scale bar = 100 µm. The data are presented as the mean ± SD of at least three independent experiments. **p* < 0.05, ***p* < 0.001 vs. the PHA-Ctrl groups; ns *p* > 0.05 represents no significance vs. PHA-Ctrl group.

Supplementary Figure 4 cHGF inhibits recipient rat aCD8^+^ cell activity in liver allografts and blood and ameliorates the inflammatory burden in liver allografts. **(A)** IF staining for CD8^+^ T cells (red) and TUNEL staining among aCD8^+^ T cells in serial 5-μm-thick liver allograft sections from allo-OLT model rats on POD 7 and quantification analysis of the percentages of aCD8^+^ T cell among DAPI+ cells (red bar) and TUNEL expression among aCD8^+^ T cells (green bar); *n* = 6. **(B)** IF staining for CD8^+^ T cells (red) in naive liver tissues (less than 4% of CD8^+^T cells, a bar chart); *n* = 3. **(C—i, ii)** FCM detection of aCD8^+^ cells in blood from the recipient rats that received either allo-OLT (i, top two panels) or cHGF (i, bottoms two panels) and analysis for the cHGF (i, red bar, *n* = 6/group), allo-OLT (i, black bar, *n* = 6/group), and cHGF plus anti-cMet-Ab (ii, green bar, *n* = 6/group on POD 7. **(D—i–iv)** Double IF staining for CD8 (red) and ki-67 (green) in liver allografts from the allo-OLT and cHGF groups on POD 7 (i) and 10–12 (ii), and the group of recipient rats received anti-cMet-Ab on the two time points (iii, iv), which were analyzed and the results are shown in (i), *n* = 6/group. **(E—i–iv)** TUNEL staining (green) of CD8^+^ cells (red) point) and anti-cMet-Ab (ii, 400 ng/mL, intrasplenic, boxes, *n* = 6/group/time point) 2 days before and during surgery and analyzed on POD7 and 10–12 (iii, iv), whose analysis is shown in , *n* = 6/group. Original magnification, ×400; scale bar = 200 µm; the boxes represent a quarter area of the whole stained field. **(F—i, ii)** H&E staining images of infiltrating cells around the PVs [(a) boxes], BDs [(b) boxes], and VEs [(c) arrows] in the cHGF and plus anti-cMet-Ab groups on POD 7 (i) and 10–12 (ii). The analyzed RAIS based on Banff’s criteria is shown in . Original magnification ×200; scale bar = 100 µm. The data are presented as the mean ± SD of at least three independent experiments. ***p* < 0.001 vs. the allo-OLT group #*p* < 0.05 vs. the cHGF group, with Student’s *t*-test.

Supplementary Figure 5 cHGF signaling inhibits recipient rat splenic aCD8^+^ cell activity. **(A—i, ii**) IF staining for CD8 (red) in serial 5-μm-thick sections in groups of allo-OLT (black bar), cHGF (red bar) (i, boxes), and anti-cMet-Ab (ii, green bar) on POD7, and their quantitative analysis. **(B—i–iv)** Analysis of cell proliferation for Ki-67 (green) and CD8 (red) data from double IF-stained sections on POD 7 (i, boxed) and 10-12 (ii, boxes) in the allo-OLT and cHGF groups and quantification (green-striped bars); the staining results in the anti-cMet-Ab-treated groups are shown in iii and iv (green bars); *n* = 6/subgroup/time point. **(C—i–iv)** Analysis of cell apoptosis by IF staining for TUNEL (green) and CD8 (red in the allo-OLT and cHGF groups on POD 7 (i, boxes) and 10–12 (ii, boxes), green-striped bars; staining and analysis of the anti-cMet-Ab group (iii, boxes, green bars); *n* = 6/subgroup/time point. Original magnification, ×400; scale bar = 200 µm. The data are presented as the mean ± SD of at least three independent experiments. ***##P* < 0.01 vs. the allo-OLT or cHGF groups.

Supplementary Figure 6 Assessment of aCD8^+^ T cell activity and its correlation with HGF in liver allografts from patients with ARJ. (**A**) A cartoon image of merged brick color of red, green, pink, and blue signals represented by an arrow. **(B—i–iv)** Assessment of acute liver rejection based on Banff’s criteria by H&E staining for infiltration cells around the PVs [(a) boxes], BDs [(b) boxes], and VEs [(c) arrows] in liver samples from patients with ARJ (ii, *n* = 6) and identified analysis with RAI scores (iii, iv, dark pink bars, *n* = 6) using HA liver samples as controls (i, iii, iv, light pink bars, *n* = 6). Original magnification ×200; scale bar = 100 µm. RAI scores measured as RAISs (0–3) (iii) and tRAISs (0–9) (iv) based on Banff’s criteria. **(C, D)** IHC staining for CD8 and HGF in liver specimens from patients with ARJ in the same field of view; *n* = 6/group. **(D)** and HA controls **(C)**, n1–n3 represent individuals, and the boxes represent one-sixth area of the whole stained field. **(E)** Double IF staining and analysis for CD8^+^ T cells (green) in total cells (DAPI+) (green bar) and TUNEL+ cells (red, merged as brown, arrow) in CD8^+^ T cells (red bar) of patients with ARJ, *n* = 6; the box represents a quarter of whole stained field. Original magnification, ×400; scale bar = 200 µm. The data are presented as the means ± SDs of at least three independent experiments. ***p* < 0.001 vs. HA liver tissues (Student’s *t*-test, two-way ANOVA).
