# Supplementary figures and images for: Comparative effects of hepatocyte growth factor and tacrolimus on acute liver allograft early tolerance
Source: Front Immunol. 2023 Aug 8;14:1162439. doi: 10.3389/fimmu.2023.1162439 (PMC10444199; doi:10.3389/fimmu.2023.1162439)

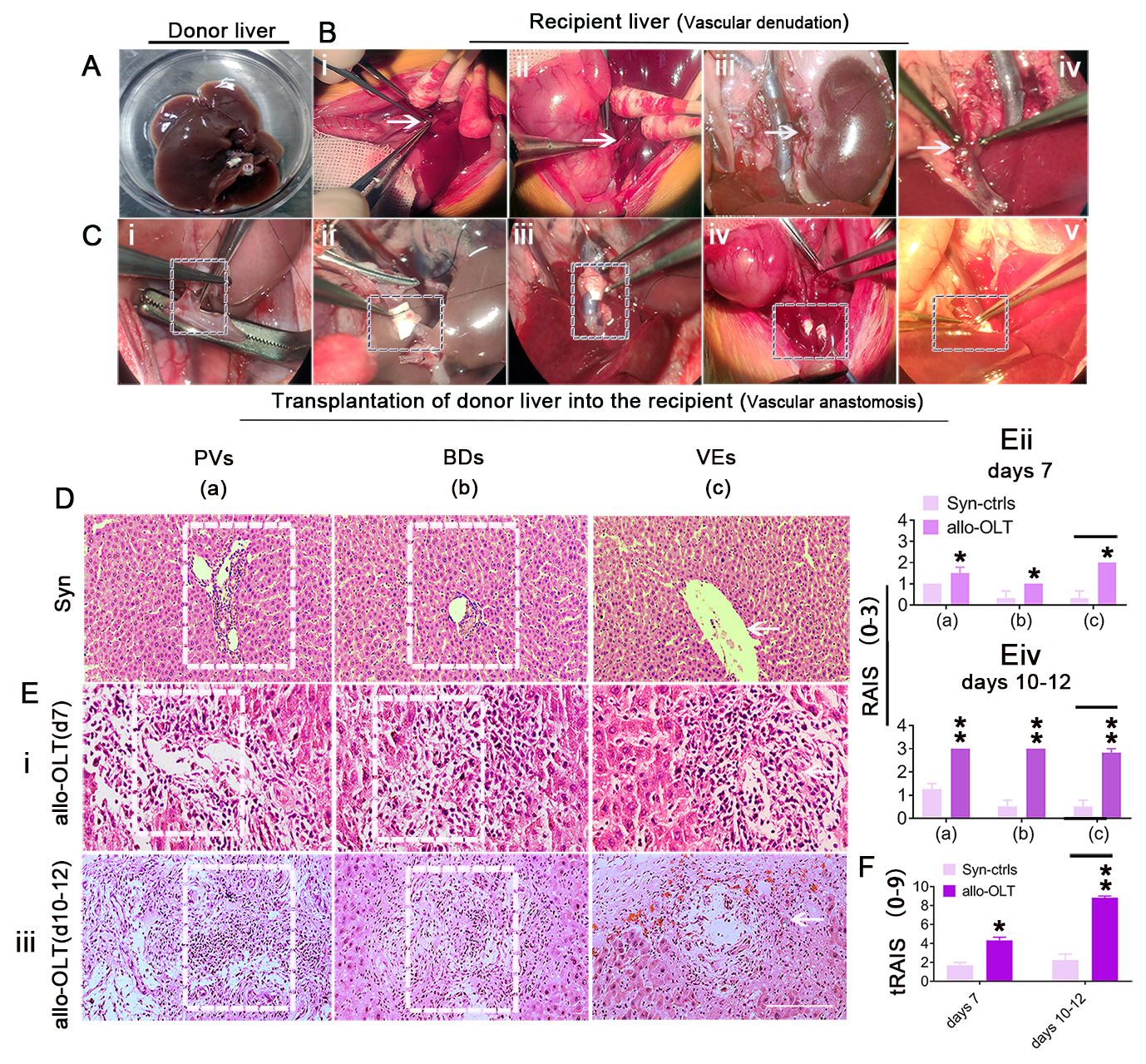

Supplement: Supplementary Figure 1 — Surgery for establishment of the DA rat-to-Lewis rat allogeneic orthotopic liver transplantation model (allo-OLT). (A) Donor liver preparation. Donor livers were removed from DA rats, flushed with 15 mL of cold saline solution (40 units/mL) and stored at 4°C until transplantation. (B—i–iv) Recipient liver preparation (vascular denudation). The PVs (i, arrow) and pyloric veins (ii, arrow) were isolated and ligated with an 8–0 suture. The right suprarenal vein (iii, arrow) was severed between the 8–0 sutures, and the liver was freed from its posterior ligaments by cutting under gentle traction. The IVC (iv, arrow) was isolated down to the left renal vein. (C—i–v) Transplantation of donor’s liver into the recipients (vascular anastomosis). The donor’s liver was placed into the abdominal cavity, and end-to-end anastomosis between the suprahepatic vena cavas (SVCs) of the graft and the recipient (i, box) was performed. The recipient’s PV was flushed with UW solution, and a cuff was inserted into the vessel. An 8–0 suture was then secured to the recipient’s PV around the cuff (ii, box). The clamps on the SVC and the PV were removed to reestablish flow. Finally, the IVC (iii, box) and BD cuffs (v, box) (iii–v) were connected with an 8–0 suture. (D) H&E staining of infiltrating cells around the PVs (a, boxes), BDs (b, boxes), and VEs (c, arrows) in the liver allografts of Syn rats on POD 7, n = 6. (E—i–iv) The same stained liver allograft serial 5-μm-thick sections (i, iii) and scored RAISs on POD 7 (ii) and 10–12 (iv), n = 6/group/time point. (F) The same staining in liver tissues from rats subjected to allo-OLT on POD 7 and 10–12 (boxes) and assessment of tRAISs from sum of RAISs, n = 6/group/time point. Original magnification = ×200; scale bar = 100 µm. The data are presented as the mean ± SD of at least three independent experiments. *p < 0.05, **p < 0.001 vs. the Syn group either on POD 7 or 10–12. [file Image_1.tif]

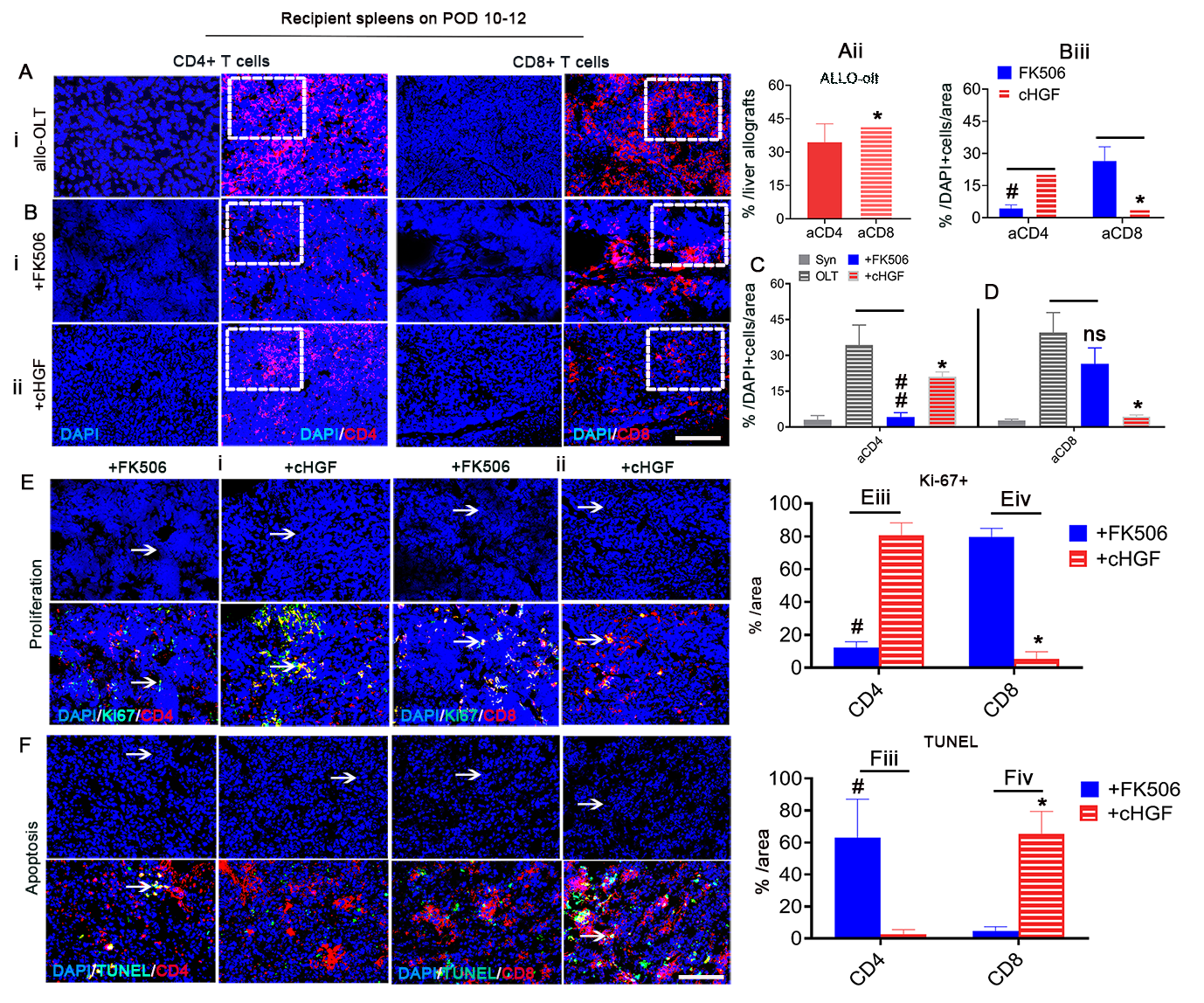

Supplement: Supplementary Figure 2 — cHGF mainly suppressed the aCD8+ T cell subpopulation, while FK506 mainly affected the aCD4+ T cell subpopulation in the recipient’s spleens. (A—i, ii) IF staining (i, boxes) for splenic aCD4+ (red) and aCD8+ (stripe) T cells in the allo-OLT group (i, boxes) and quantitative analysis (ii). (B—i–iii) The same staining for aCD4+ (red) and aCD8+ (red) T cells of the rats that received FK506 (i, boxes) and cHGF (ii, boxes) and comparative analysis of the proportions of CD4+ and CD8+ T cells between the FK506 (blue bar) and cHGF (red-striped bar) groups (iii); n = 6. (C, D) Analysis of recipient aCD4+ T cells (C) and aCD8+ T cells (D) in the Syn (gray) and allo-OLT (gray stripe) groups following FK506 (blue) and cHGF (red stripe) treatment; n = 6/group; the boxes represent a quarter area of the whole stained field. (E—i–iv) Double IF staining for Ki-67 (green) (arrows) within aCD4+ (red, i, iii) and CD8+ T cells (red, ii, iv) and comparative analysis of the positive cell numbers in the T cell subpopulations from the tissue sections of the rats treated with FK506 (blue bars) and cHGF (red striped bars). (F—i–iv) TUNEL staining (arrows) of aCD4+ (i, iii) and aCD8+ (ii, iv) T cells and comparative analysis of the same tissue sections treated with FK506 (blue bars) and cHGF (red-striped bars). Original magnification = ×400; scale bar = 200 µm. The data are presented as the mean ± SD of at least three independent experiments. *p < 0.05 vs. either the allo-OLT or FK506 group; *#p < 0.05 vs. the CD4+T cells or FK506 or cHGF group; ##p < 0.001 vs. the allo-OLT group; ns p > 0.05. [file Image_2.tif]

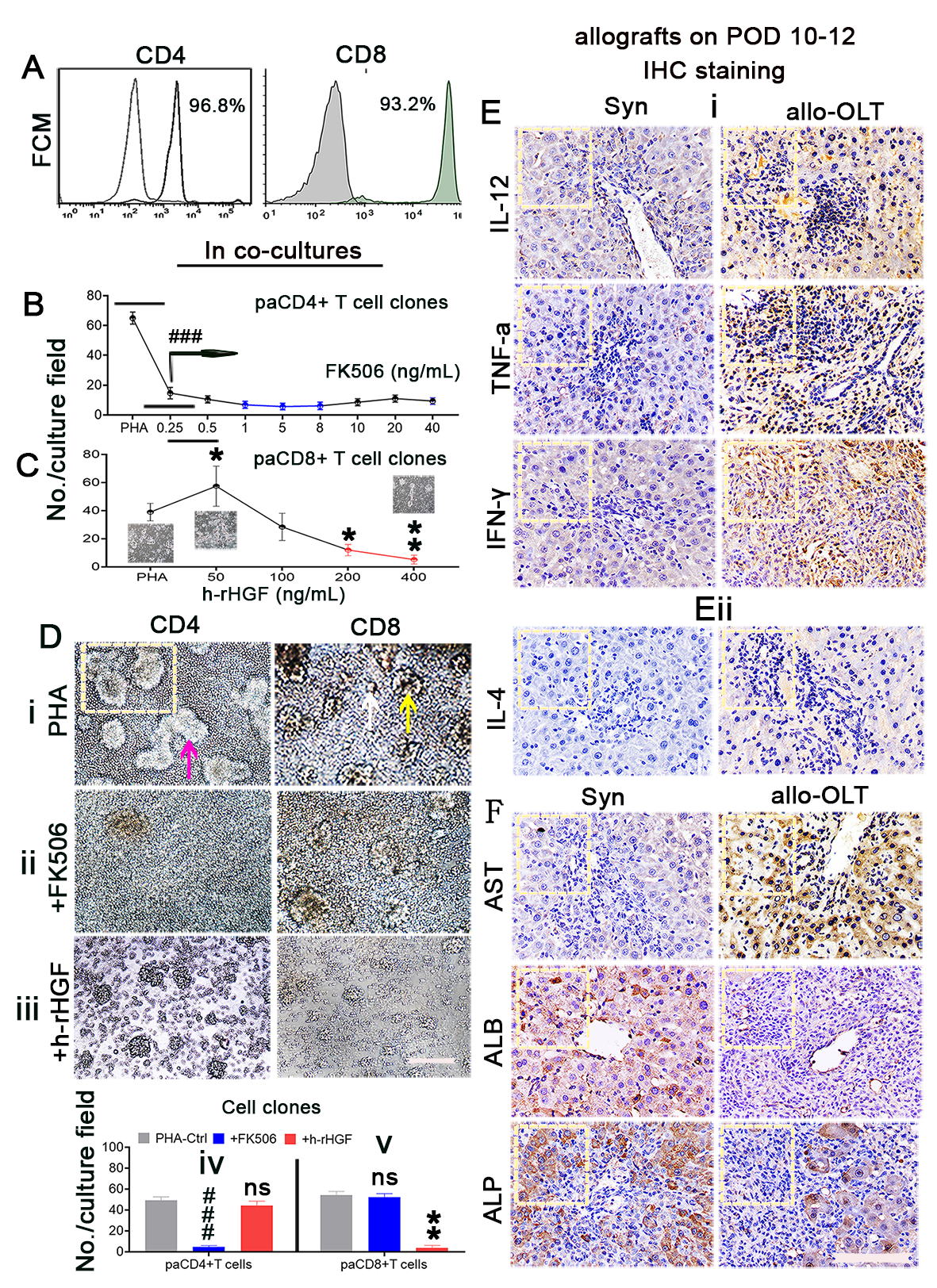

Supplement: Supplementary Figure 3 — Treatment of recipient aCD4+ and aCD8+ T cells with FK506 and h-rHGF in cocultures and analysis of cytokines and hepatic proteins in liver allografts by IHC staining. (A) FCM analysis of MACS-purified splenic aCD4+ T cells and aCD8+ T cells; n = 3. (B) A total of 1 × 10^5^ of the CD4+ T cells were treated with FK506 at 0.25–40 ng/mL for 24 h; all concentrations resulted in these cells’ suppression compared with PHA-stimulated aCD4+ T cells (as controls), and 1–8 ng/mL (blue line) was selected for the coculture studies. (C) The same number of aCD8+ T cells as aCD4+ T cells were treated with h-rHGF at different concentrations for 24 h, and ≤50 ng/mL h-rHGF significantly promoted sphere growth (an image represents more spheres vs. PHA-Ctrl, an image); ≥200 ng/mL, h-rHGF greatly (*p < 0.05) decreased the number of spheres compared with the PHA control; at 400 ng/mL, the results were even more significant (**p < 0.001, an image) compared with the PHA-Ctrl; thus, 400 ng/mL h-rHGF was selected for the in vitro clonal expansion study. (D—i–v) 0.5-1% PHA stimulated the CD4+ and CD8+ T cells (i) treated with FK506 (1–8 ng/mL) (ii) or h-rHGF (iii) for 24 h in cultures; then, expanded clonal numbers were counted from small, middle, to large as arrows of pink, yellow, and white, respectively, and quantitative analysis was separately performed for CD4 (iv, FK506: blue bars) and CD8 (v, h-rHGF: red bars); the box represents one-half area of the whole stained field. (E—i, ii) IHC staining for the proinflammatory cytokines IL-12, TNF-α, and IFN-γ (i, boxes) and anti-inflammatory cytokine IL-4 (ii, boxes) and their quantification analysis in shown as gray and black bars. (F) IHC staining of liver allograft sections for the hepatic proteins AST, ALB, and ALP from the Syn and allo-OLT groups (boxes); n = 6/protein/cytokine, and their quantification analysis is shown in as gray and black bars; the boxes represent one-sixth area of the whole stained field. Original magnification = ×200; sc [file Image_3.tif]

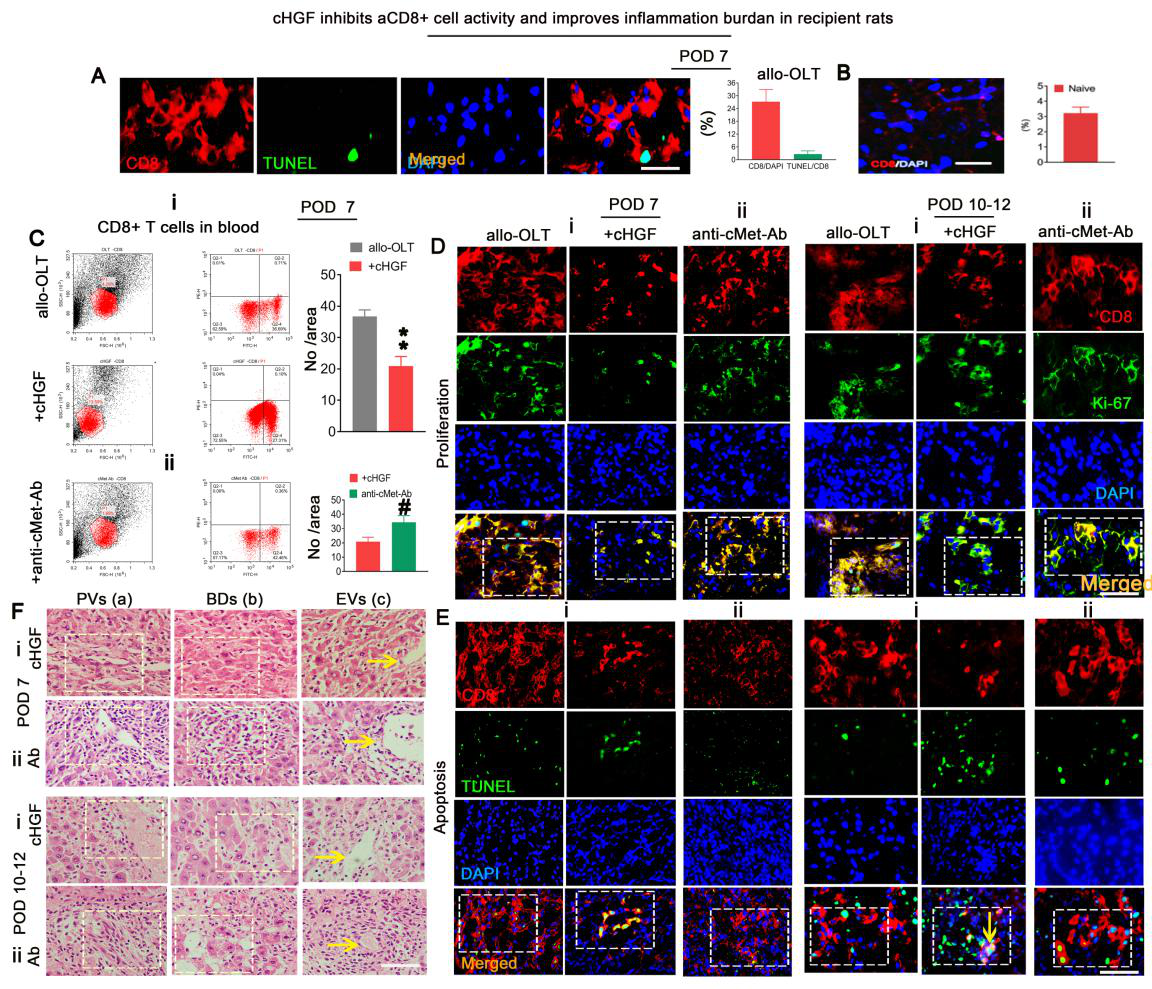

Supplement: Supplementary Figure 4 — cHGF inhibits recipient rat aCD8+ cell activity in liver allografts and blood and ameliorates the inflammatory burden in liver allografts. (A) IF staining for CD8+ T cells (red) and TUNEL staining among aCD8+ T cells in serial 5-μm-thick liver allograft sections from allo-OLT model rats on POD 7 and quantification analysis of the percentages of aCD8+ T cell among DAPI+ cells (red bar) and TUNEL expression among aCD8+ T cells (green bar); n = 6. (B) IF staining for CD8+ T cells (red) in naive liver tissues (less than 4% of CD8+T cells, a bar chart); n = 3. (C—i, ii) FCM detection of aCD8+ cells in blood from the recipient rats that received either allo-OLT (i, top two panels) or cHGF (i, bottoms two panels) and analysis for the cHGF (i, red bar, n = 6/group), allo-OLT (i, black bar, n = 6/group), and cHGF plus anti-cMet-Ab (ii, green bar, n = 6/group on POD 7. (D—i–iv) Double IF staining for CD8 (red) and ki-67 (green) in liver allografts from the allo-OLT and cHGF groups on POD 7 (i) and 10–12 (ii), and the group of recipient rats received anti-cMet-Ab on the two time points (iii, iv), which were analyzed and the results are shown in (i), n = 6/group. (E—i–iv) TUNEL staining (green) of CD8+ cells (red) point) and anti-cMet-Ab (ii, 400 ng/mL, intrasplenic, boxes, n = 6/group/time point) 2 days before and during surgery and analyzed on POD7 and 10–12 (iii, iv), whose analysis is shown in , n = 6/group. Original magnification, ×400; scale bar = 200 µm; the boxes represent a quarter area of the whole stained field. (F—i, ii) H&E staining images of infiltrating cells around the PVs [(a) boxes], BDs [(b) boxes], and VEs [(c) arrows] in the cHGF and plus anti-cMet-Ab groups on POD 7 (i) and 10–12 (ii). The analyzed RAIS based on Banff’s criteria is shown in . Original magnification ×200; scale bar = 100 µm. The data are presented as the mean ± SD of at least three independent experiments. **p < 0.001 vs. the allo-OLT group #p < 0.05 vs. the cHGF group, with Student’s t-tes [file Image_4.tif]

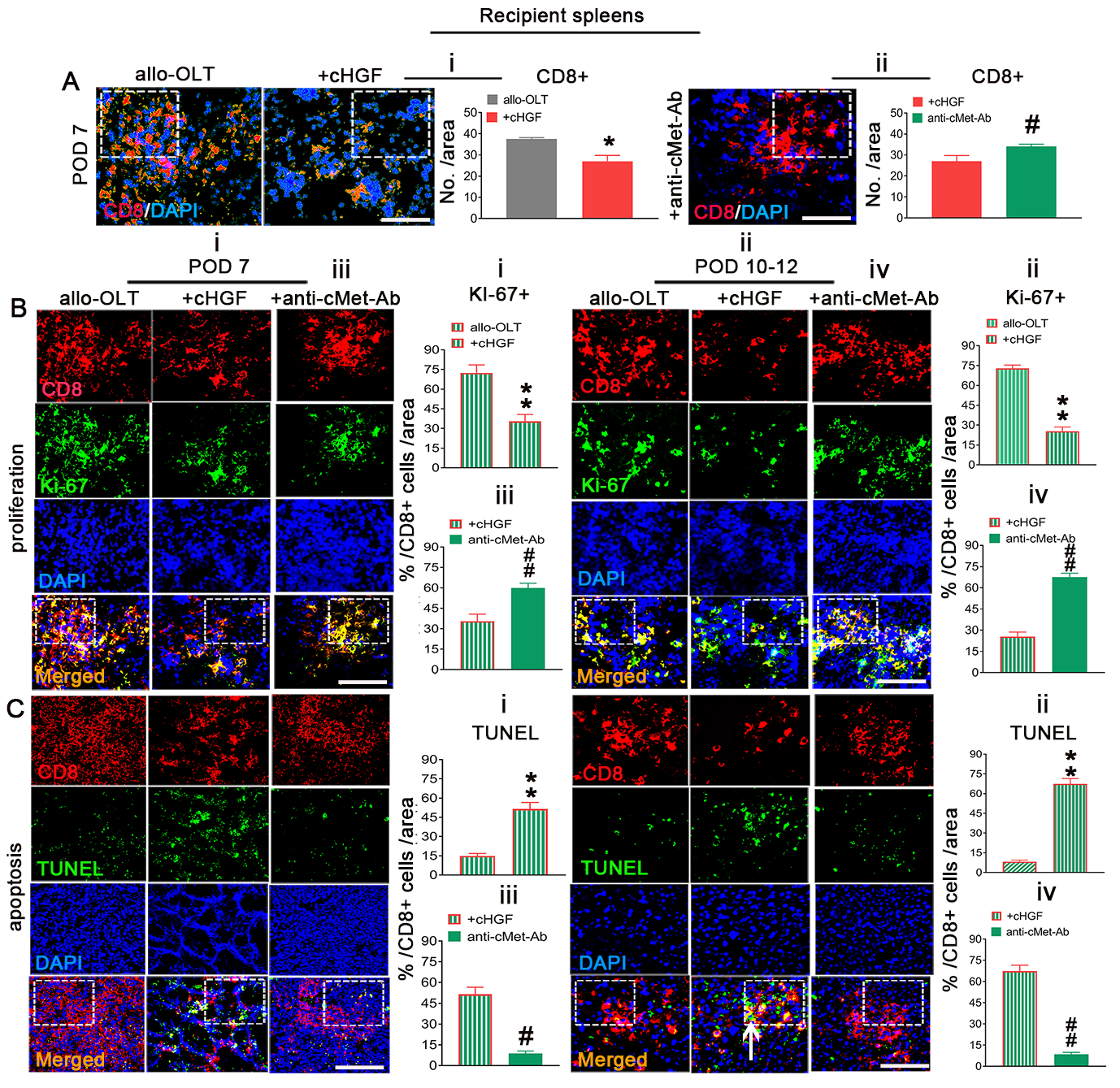

Supplement: Supplementary Figure 5 — cHGF signaling inhibits recipient rat splenic aCD8+ cell activity. (A—i, ii) IF staining for CD8 (red) in serial 5-μm-thick sections in groups of allo-OLT (black bar), cHGF (red bar) (i, boxes), and anti-cMet-Ab (ii, green bar) on POD7, and their quantitative analysis. (B—i–iv) Analysis of cell proliferation for Ki-67 (green) and CD8 (red) data from double IF-stained sections on POD 7 (i, boxed) and 10-12 (ii, boxes) in the allo-OLT and cHGF groups and quantification (green-striped bars); the staining results in the anti-cMet-Ab-treated groups are shown in iii and iv (green bars); n = 6/subgroup/time point. (C—i–iv) Analysis of cell apoptosis by IF staining for TUNEL (green) and CD8 (red in the allo-OLT and cHGF groups on POD 7 (i, boxes) and 10–12 (ii, boxes), green-striped bars; staining and analysis of the anti-cMet-Ab group (iii, boxes, green bars); n = 6/subgroup/time point. Original magnification, ×400; scale bar = 200 µm. The data are presented as the mean ± SD of at least three independent experiments. **##P < 0.01 vs. the allo-OLT or cHGF groups. [file Image_5.tif]

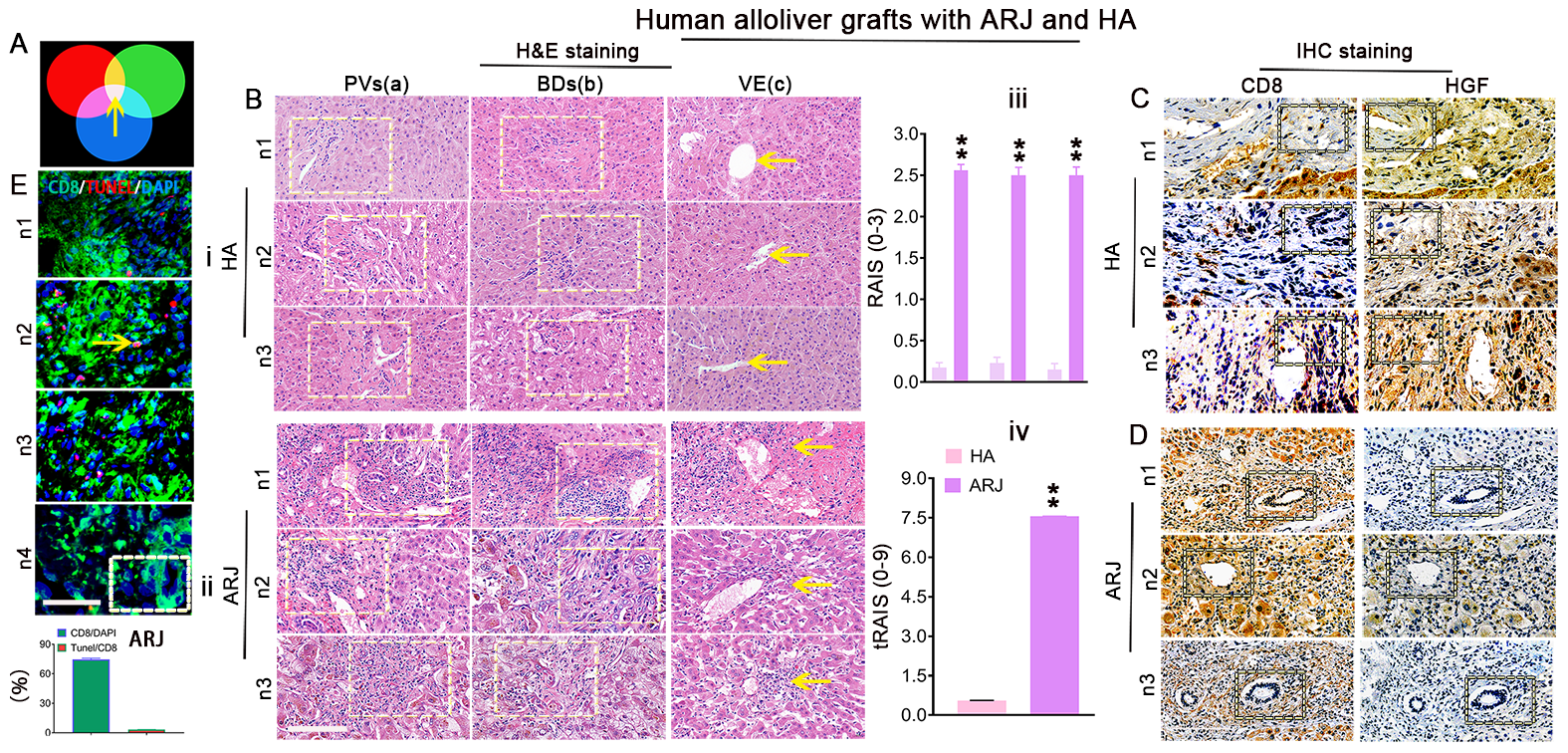

Supplement: Supplementary Figure 6 — Assessment of aCD8+ T cell activity and its correlation with HGF in liver allografts from patients with ARJ. (A) A cartoon image of merged brick color of red, green, pink, and blue signals represented by an arrow. (B—i–iv) Assessment of acute liver rejection based on Banff’s criteria by H&E staining for infiltration cells around the PVs [(a) boxes], BDs [(b) boxes], and VEs [(c) arrows] in liver samples from patients with ARJ (ii, n = 6) and identified analysis with RAI scores (iii, iv, dark pink bars, n = 6) using HA liver samples as controls (i, iii, iv, light pink bars, n = 6). Original magnification ×200; scale bar = 100 µm. RAI scores measured as RAISs (0–3) (iii) and tRAISs (0–9) (iv) based on Banff’s criteria. (C, D) IHC staining for CD8 and HGF in liver specimens from patients with ARJ in the same field of view; n = 6/group. (D) and HA controls (C), n1–n3 represent individuals, and the boxes represent one-sixth area of the whole stained field. (E) Double IF staining and analysis for CD8+ T cells (green) in total cells (DAPI+) (green bar) and TUNEL+ cells (red, merged as brown, arrow) in CD8+ T cells (red bar) of patients with ARJ, n = 6; the box represents a quarter of whole stained field. Original magnification, ×400; scale bar = 200 µm. The data are presented as the means ± SDs of at least three independent experiments. **p < 0.001 vs. HA liver tissues (Student’s t-test, two-way ANOVA). [file Image_6.tif]
